# Understanding Communication Signals during Mycobacterial Latency through Predicted Genome-Wide Protein Interactions and Boolean Modeling

**DOI:** 10.1371/journal.pone.0033893

**Published:** 2012-03-20

**Authors:** Shubhada R. Hegde, Hannah Rajasingh, Chandrani Das, Sharmila S. Mande, Shekhar C. Mande

**Affiliations:** 1 Structural Biology Laboratory, Centre for DNA Fingerprinting and Diagnostics, Gruhakalpa, Nampally, Hyderabad, India; 2 Bio-Sciences R & D Division, TCS Innovation Labs, Tata Consultancy Services, Hyderabad, India; University of Delhi, India

## Abstract

About 90% of the people infected with *Mycobacterium tuberculosis* carry latent bacteria that are believed to get activated upon immune suppression. One of the fundamental challenges in the control of tuberculosis is therefore to understand molecular mechanisms involved in the onset of latency and/or reactivation. We have attempted to address this problem at the systems level by a combination of predicted functional protein∶protein interactions, integration of functional interactions with large scale gene expression studies, predicted transcription regulatory network and finally simulations with a Boolean model of the network. Initially a prediction for genome-wide protein functional linkages was obtained based on genome-context methods using a Support Vector Machine. This set of protein functional linkages along with gene expression data of the available models of latency was employed to identify proteins involved in mediating switch signals during dormancy. We show that genes that are up and down regulated during dormancy are not only coordinately regulated under dormancy-like conditions but also under a variety of other experimental conditions. Their synchronized regulation indicates that they form a tightly regulated gene cluster and might form a latency-regulon. Conservation of these genes across bacterial species suggests a unique evolutionary history that might be associated with *M. tuberculosis* dormancy. Finally, simulations with a Boolean model based on the regulatory network with logical relationships derived from gene expression data reveals a bistable switch suggesting alternating latent and actively growing states. Our analysis based on the interaction network therefore reveals a potential model of *M. tuberculosis* latency.

## Introduction

Increased rate of tuberculosis (TB) infection and the emergence of multi and extensively-drug-resistant tuberculosis [1] have necessitated urgent efforts towards enhanced understanding of its causative agent, *Mycobacterium tuberculosis*. The complete genome sequence of the common laboratory strain *M. tuberculosis* H37Rv was unraveled in 1998 [2] and the sequence was later re-annotated [3]. The genome annotation combined with comparative genomic studies has revealed several novel features including gene families that are unique to this bacterium such as PE and PPE genes and the eukaryotic like serine threonine protein kinases (STPKs). The genome also harbors unique genes involved in lipid biosynthesis, drug resistance and pathogenesis [2].

Owing to its distinct characteristics and the disease causing ability, *M. tuberculosis* has been studied widely across the world. For example, different experiments have accumulated information about the biochemical and structural properties of a number of proteins of this organism [4], [5]. There is also large amount of data on mycobacterial gene expression under a variety of growth conditions [6]. Genome-wide surveys of essential genes of *M. tuberculosis*, both *in vitro* and those potentially involved in pathogenesis, have been carried out [7]–[9]. Similarly, large-scale proteome profiling to classify *M. tuberculosis* proteins into different cellular compartments has been carried out [10]. Such varied studies have helped in understanding not only the unique genetic makeup of the bacillus, but also the possible roles of individual genes during different steps of pathogenesis.

*M. tuberculosis* has also been the centre of attention for a few studies executed at the systems level. In this regard, there have been attempts to model gene regulation, protein interactions and metabolic pathways of the organism. Functional linkage maps of *M. tuberculosis* have been defined by using genome context methods [11]. In another study, Balaszi et al have assembled gene regulation information and studied transcriptional changes that might mediate switch to dormancy [12]. Flux balance analysis (FBA) on the model of mycolic acid biosynthesis pathway has revealed potential drug targets pertaining to this pathway [13]. Nonetheless, the systems level understanding of *M. tuberculosis* remains inadequate. One of the major obstacles being that a large fraction of genes are either putative or are unannotated, and thereby coherence among different pathways that contribute to virulence remains to be defined systematically. There is therefore a pressing need to integrate different approaches to understand tuberculosis and perceive mechanisms by which *M. tuberculosis* enters a dormant phase, or emerges out of it. A new promise in this aspect is the availability of a number of genome sequences of clinical strains and the data gathered by individual and high-throughput experiments which permit studies at the systems level.

One of the promising approaches to understand complex functional associations of the molecules and their organization is by analyzing genome-wide protein∶protein interactions by means of graph theoretical representations. In this regard, there have been a few attempts to generate protein interaction maps of *M. tuberculosis*, all of them being *in silico* predictions. Strong et al used genome context methods to derive a functional linkage map of 4,886 interactions among 1,958 proteins followed by clustering of the network in order to reconstruct some of the biochemical pathways in *M. tuberculosis* [11]. Another study involved translating high confidence interactions of *E. coli* to *M. tuberculosis* [14]. A set of 6,091 interactions among 793 proteins was obtained in this study to identify proteins involved in signaling pathways. The interaction database STRING [15] houses protein interactions of *M. tuberculosis* derived by literature curation and other methods. The highest confidence interactions in STRING which are supported by multiple methods or curation include around 6,403 interactions between 1,653 proteins. However, none of the above sources represents a comprehensive collection of *M. tuberculosis* protein interactions.

We have attempted to combine genome context methods, namely phylogenetic profile [16], [17], gene distance [18] operonic co-occurrence [19], [20] with the available high throughput gene expression studies for the prediction of genome-wide functional linkages. The predictive features of these were combined using a Support Vector Machine (SVM). These functional linkages were analyzed to understand the phenomenon of latency, thus providing an integrated perspective of latency. In addition, a Boolean model is employed to investigate the patterns of expression of genes identified as being up and down-regulated during latency. Boolean or logical models [21] are known to be an effective and straightforward means of reproducing the behaviour of gene regulatory networks over time [22]–[24], especially for systems where only qualitative data is available. The model simulations strengthen our understanding of the regulatory control exerted by a small number of genes in order to sustain the latency stage.

## Results

### Generation of the Protein Functional Linkages

We have reported earlier that the genome context methods, namely phylogenetic profile, operonic frequency and gene distance can be effectively combined using a SVM to predict protein functional linkages in *E. coli* [25]. Apart from the genome context methods it is interesting to note that the proteins which have functional relations are also known to show good correlation in their gene expression [26]. We therefore attempted to include correlation in gene expression among the genes as a feature for SVM training.

**Figure 1:**
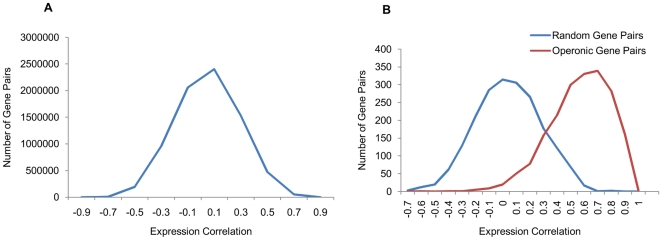
Pearson correlation coefficient between expression values of pairs of genes. A) The genome-wide gene expression correlations of all the gene pairs and B) expression correlation between operonic gene pairs and randomly paired non-operonic gene pairs. As anticipated, it is evident that the genes on operons exhibit higher correlation in their expression compared to the non-operonic gene pairs.

It is well known that large-scale gene expression datasets are expected to be noisy [27]. However, the expression levels between gene pairs, if functionally related, are likely to be correlated across different experimental conditions. In general, no gene pair is anticipated to exhibit high expression correlation except when the two genes are coregulated thereby suggesting a functional relationship between the two. This conjecture was sought to be tested using known operonic gene pairs. Significantly, the operonic gene pairs show high expression correlation compared to the same number of randomly generated non-operonic pairs (Figure 1). This observation, therefore, strengthened the confidence in using the microarray data for interaction predictions. Thus, we have supplemented genome context methods with correlations in gene expression to generate genome-wide protein functional linkages map of *M. tuberculosis*.

**Figure 2:**
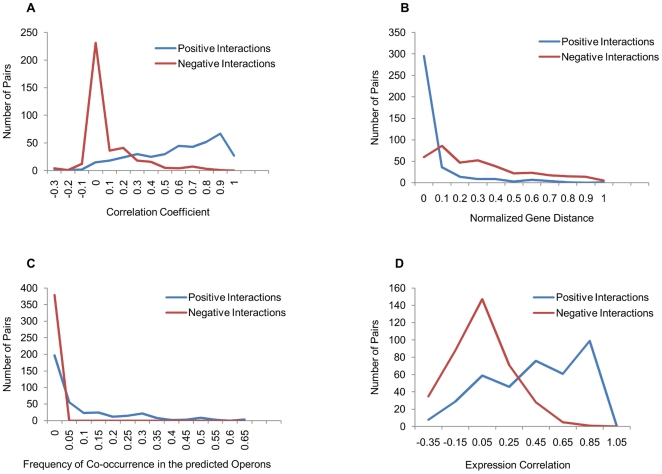
Plots indicating the distinctive behavior of the positive and negative protein interaction pairs with respect to genome context methods and gene expression correlations. All the four features show statistically significant and distinct distribution for the positive and negative pairs. A) Phylogenetic Profile, B) Gene Distance, C) Operonic Method and D) Expression Correlation.

In order to test whether the positive and negative pairs used in training the SVM show characteristic distribution for the data features chosen, students' t-test was performed on the available positive pairs and equal number of randomly chosen negative pairs. Figure 2 depicts the distribution of vectors of the gene pairs used in training, which show a distinctly different distribution with a p value of 2.2e^−16^. All the data features chosen are therefore capable of distinguishing between positive and negative pairs, suggesting their potential application in the prediction of functional linkages. Thus, using these features, optimization of training for the SVM was performed with 5-fold cross-validation. Furthermore, performance of SVM classification was evaluated by plotting ROC (Figure S1), and the best predictive model showed AUC of 0.834 with 0.85 Mathews Correlation Coefficient (MCC). This optimized model with prediction accuracy and sensitivity of 88% and 76% respectively, was chosen for prediction of functional linkages on the genome-wide scale.

### The Functional Linkages of *M. tuberculosis* Proteins

The predicted protein functional linkages network has 32,546 interactions among 3,571 proteins (Table S1). The largest connected component of the network comprises of 95% of the nodes and has a diameter of 12. The network shows scale free property with the degree exponent of 1.67. The overall topological parameters of the network are summarized in Table 1. Comparison of the network with previously derived interaction maps shows around 30% overlap (Table S2). Though the overlap appears less, it is observed that protein interaction maps obtained from different sources generally have fewer interactions in common due to inherent bias in the method used or the noise [25].

**Table 1:** Topological Properties of the predicted protein functional linkages.

Number of Interactions	32546
Number of Nodes	3571
Percentile Core Nodes	95%
Average Degree	19.2
Degree Exponent	1.67
Diameter	12
Average Clustering Coefficient	0.22

**Figure 3:**
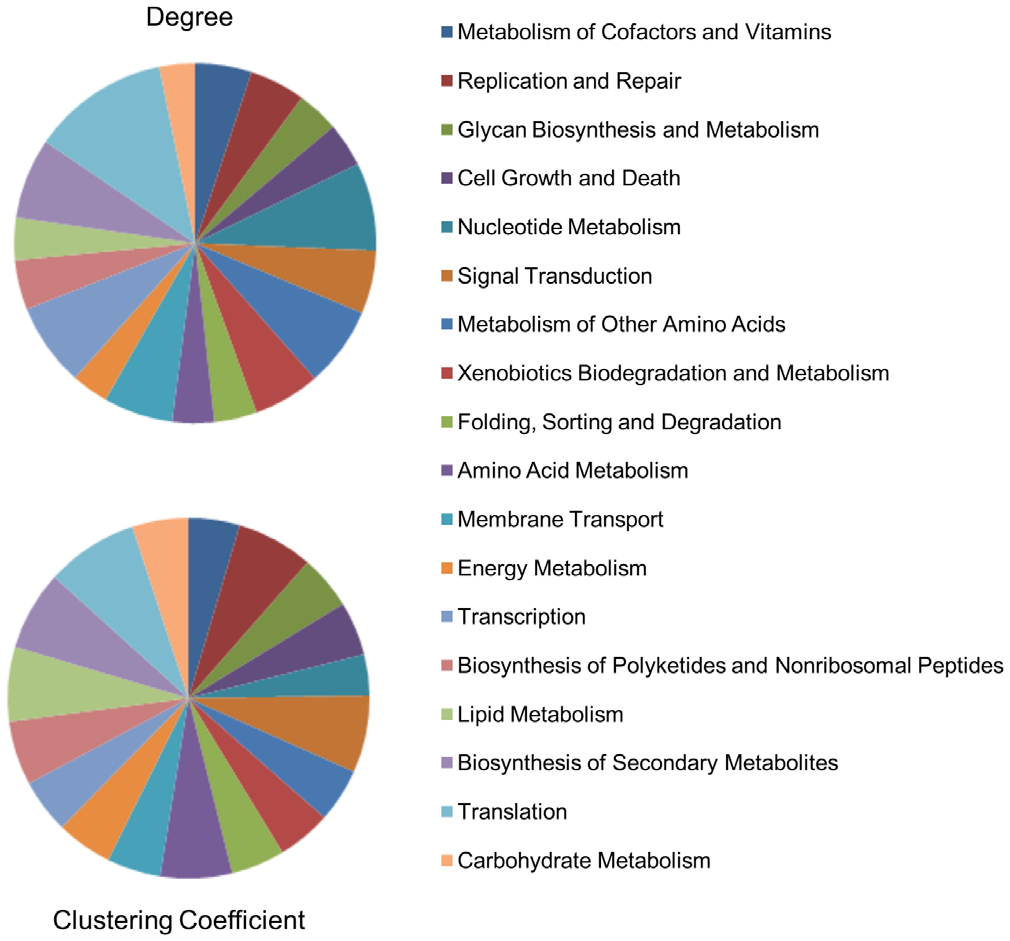
Average clustering coefficient and degree of the proteins across metabolic pathways. Proteins show high degree or high clustering coefficient depending on their function. Translation pathway proteins have high degree as well as high clustering coefficient whereas membrane transport proteins are highly clustered with less connectivity.

The average degree and clustering coefficient of the proteins classified into different metabolic pathways is shown in Figure 3. Proteins belonging to translation pathway, such as the ribosomal proteins and other translation related proteins, have high degree as well as high clustering coefficient. On the other hand, proteins of the “cell growth and death” pathway have high degree but are less clustered. The membrane transport proteins on the other hand show less degree but are highly clustered. Thus, we observe varied relationships between clustering and degree in different metabolic pathways.

**Figure 4:**
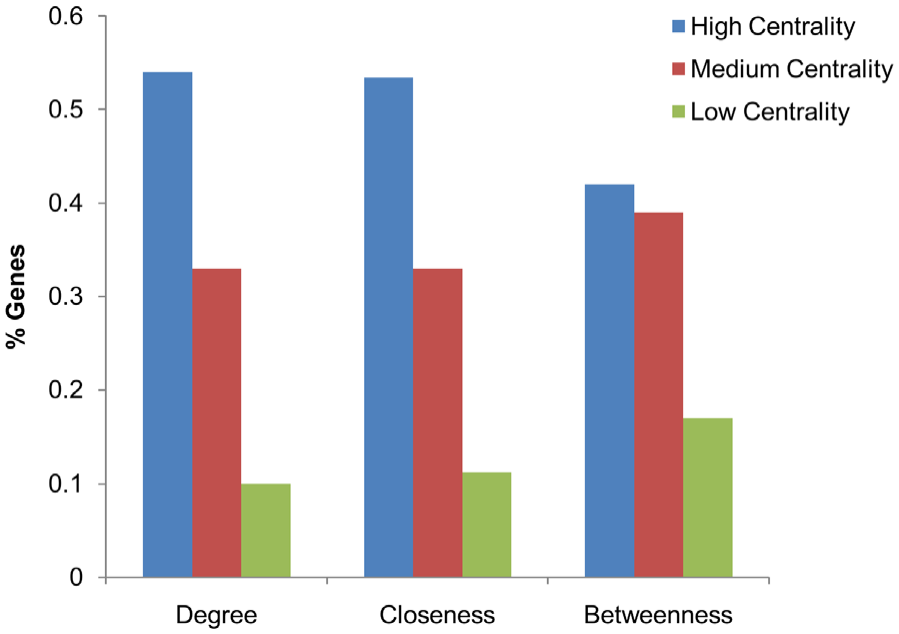
Proportion of Essential Genes in the bins of decreasing centrality values. For all the three centrality values calculated, namely degree, closeness and betweenness, it is clearly seen that there exists a good correlation between centrality and lethality.

Proteins coded by essential genes in biological networks are known to exhibit high network centrality measures compared to their counterparts [28]. This observation was tested in *M. tuberculosis* network for the experimentally proposed essential genes [7]. From our functional interaction network, we calculated three centrality values, namely degree, closeness and betweenness, for each of the genes, and divided them into three categories: high centrality (top 30% nodes), medium centrality (between 30–70% nodes) and low centrality (others). The proportion of essential genes for these three centrality parameters was plotted as shown in Figure 4. It is evident from the figure that there is a strong correlation between network centrality and gene essentiality in our proposed network. The proteins of information pathway such as DnaG, DnaB, Rho and ribosomal proteins; proteins belonging to intermediary metabolism and respiration such as ATP synthase subunits, purine and pyrimidine biosynthesis proteins; proteins in the amino acid biosynthesis pathways and cell division proteins such as FtsX, FtsZ and FtsH; and proteins of cell wall formation such as MurA, MurB, MurC and MurD show very high network centrality values. Interestingly, PPE4 and PPE46 of the PPE family proteins have high centrality values, suggesting that these proteins might also be constituents of the essential gene set of *M. tuberculosis*. The complete list of proteins with high centrality values is given in Table S3.

Proteins involved in closely related functions, or those involved in the same biochemical pathway, are known to cluster together to form functional units in an interaction map [29]. Clustering of our network indicated that it is divided into 184 clusters which we have annotated using the pathway information taken from Sanger and TubercuList databases (Table S4). The largest cluster is enriched with cell wall and cell processes, virulence related proteins and a large number of conserved hypothetical proteins. The ribosomal proteins and other translation related proteins cluster along with DNA replication and repair proteins. A large number of PE-PPE proteins were found to be associated with virulence proteins. It is likely therefore that these proteins play an important role in virulence determination, as has been suggested earlier [2], [30]. Thus, the sub-division of the interaction map into clusters based on the nature of their interaction might lead to a better understanding of cross-talks between different functional categories.

### Persistence in *M. tuberculosis*

One of the least understood phenomena associated with *M. tuberculosis* is the switch between latent and actively replicating phases. It is interesting to address this phenomenon of latency in *M. tuberculosis* through the analysis of functional interaction network. The predicted interaction map was therefore used to demarcate the proteins that might play important role in the dormant phase of *M. tuberculosis*. Towards addressing these questions, we considered several experimental studies that have attempted to simulate the dormancy phase using the *in-vitro* models to generate gene expression profiles. Such conditions include hypoxia [31]–[35], NO treatment [36], stationary phase [35] and nutrient deprivation [37]. Along with the *in-vitro* data, murine models of *M. tuberculosis* dormancy have also been studied [38]–[39]. Since such individual experiments might not capture all the dormancy features of *M. tuberculosis*, and moreover considering that the microarray experiments might be affected by intrinsic noise, we considered the commonality among these to identify key genes regulating latency.

The gene expression datasets of different persistence models of *M. tuberculosis* were used to identify up and down-regulated genes during early persistence. The list of different models and the number of up and down-regulated genes are detailed in Table S5. Intriguingly, the overlap among the differentially regulated genes between different models of *M. tuberculosis* dormancy is not very high. For example, murine model of *M. tuberculosis* latency has 7%, 19%, 6% and 3% overlap with the other available models of NO treatment, hypoxia, stationary phase growth and starvation model respectively (Table S6). We therefore considered only the genes that are common to at least 5 of the 12 expression conditions related to latency. Using this criterion, we were able to identify 50 genes that show increase in expression levels and 34 genes that are down-regulated during latency (Table S7).

In order to understand if the factors leading to dormancy are shared by different prokaryotic species we first probed evolutionary conservation of the 84 genes by constructing a binary phylogenetic profile of these genes across 481 genomes and then counted the numbers of genomes harboring these genes. Interestingly, the 50 genes that are upregulated in *M. tuberculosis* dormancy are far less conserved across species than the 34 genes that are downregulated. As few as 16 upregulated genes are present in less than 145 of the 481 genomes. On the other hand, as many as 26 down regulated genes are present in at least 336 genomes (Figure S2). The downregulated genes represent those involved in basic cellular processes, such as replication, transcription and translation. The rates of these basic cellular processes being significantly slowed during dormancy offers a possible explanation that the downregulated genes are common to many species. The remarkable observation, however, that the upregulated genes are less conserved than the downregulated genes suggests a possible unique mechanism of dormancy adapted by *M. tuberculosis*. The 50 upregulated genes therefore appear to constitute a “dormancy signal” that is unique to *M. tuberculosis*. Such a dormancy signal might then be transmitted to the genes involved in basic cellular processes in order to slow down the overall metabolic rates.

**Figure 5:**
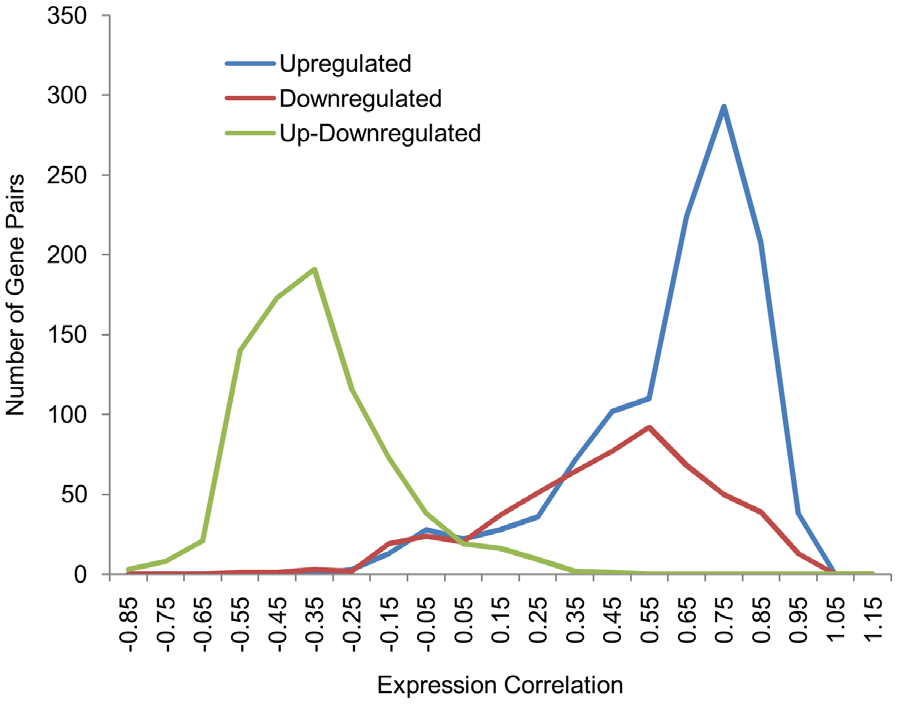
Expression Correlation between up and downregulated genes. Upregulated genes correlate in their expression and a similar trend is observed for downregulated genes. However, there is inverse correlation in expression between up and downregulated genes.

Although the 84 genes showed coordinated regulation during latency-like conditions, it was interesting to probe if similar regulation controlled their expression even under other experimental conditions. The expression correlation among the 50 upregulated genes and the 34 downregulated genes across 154 growth conditions was then computed. Interestingly, the expression values among the 50 upregulated genes correlate highly (Figure 5). Similarly, those among the 34 downregulated genes also show strong correlation. On the other hand, the up and the down-regulated genes exhibit inverse correlation. This observation suggests that the 84 genes are coordinately regulated not only under latency-like conditions, but also are regulated in a controlled manner under most other conditions of growth. The 84 genes thus form a regulon-like structure with an extensive cross talk in their expression. Such a crosstalk can possibly be uncovered using the functional interaction network.

**Figure 6:**
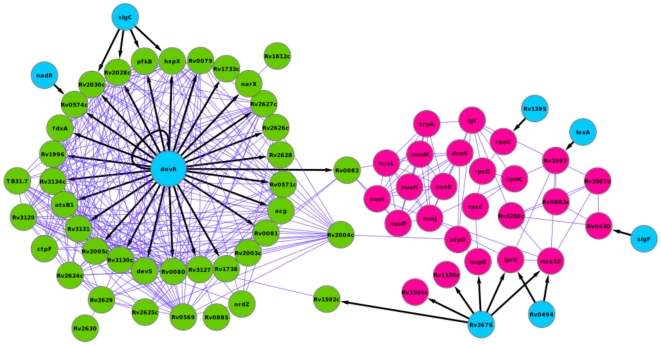
Graphical representation of functional interactions and regulatory interactions of up and downregulated genes. DosR acts as a master regulator, regulating the expression of a number of upregulated genes. A probable oxidoreductase Rv0082 under the regulation of DosR associates with the subunits of aerobic respiratory machine NADH oxidoreductase complex. Upregulated proteins are colored in green, downregulated proteins are colored in red and transcription factors are colored in blue. Directed edge denotes gene regulation and undirected edge is a protein functional linkage.

We then integrated our protein functional linkages with the available gene regulatory information in order to understand the crosstalk between the 84 latency-related genes [12]. One of the important observations upon such an integration of the two networks was that DosR, a well studied dormancy associated protein, regulates the expression of about 25 upregulated genes (Figure 6). Further, the genes regulated by DosR also interact extensively among themselves forming a clique-like architecture in the network. Moreover, important latency genes such as *hspX*, *pfkB*, *Rv2030c* and *Rv2028c* are additionally regulated by sigma factor SigC, implying a multifarious regulatory circuit of latency. On the other hand, Rv3676, a transcriptional regulatory protein of the cAMP receptor protein (CRP) family [40], regulates the expression of downregulated genes such as *Rv1566c*, *Rv1158c*, *hupB*, *lprK* and *mce1D*. Thus, a central regulatory circuit controlled by DosR, with degeneracy offered by transcription factors such as SigC and CRP, combined with an extensive network of interactions among the 84 genes appears to control latency in *M. tuberculosis*.

An interesting outcome of the network analysis pertains to the dormany signal detected by the 50 upregulated genes, and its transmission to the 34 downregulated genes. DosR interacts with two-component sensory kinases DosS (Rv3132c), DosT (Rv2027c) and Rv0845. It has been reported previously that DosR acts as a cognate response regulator of both DosS and DosT, which sense hypoxia and NO [41]. Interestingly, a classification based on the region around phosphorylated sensor kinases of *M. tuberculosis* assigns DosS, DosT and Rv0845 to the same class [42]. Whereas Rv0845 is a possible nitrate/nitrite sensor protein, DosS and DosT function as redox and hypoxia sensors respectively [43]. It is therefore likely that DosS, DosT and Rv0845 sense the dormancy signal and trigger a regulatory cascade controlled by DosR.

The gene regulatory network also suggests that a hypothetical oxidoreductase, Rv0082, is regulated by DosR with increase in its expression level under dormancy conditions. The functional interaction network further suggests that Rv0082 might associate with three of the subunits of NADH-ubiquinone oxidoreductase complex (NDH-1). The subunits of NDH-1 complex are involved in respiration and not surprisingly form a clique in the functional interaction network. Moreover, these are downregulated during latency. The expression correlation between NDH-1 genes spanning from *Rv3145–Rv3158* and ATP synthase genes spanning from *Rv1304–Rv1311* are correlated in their expression (Figure S3). This suggests a succeeding downregulation of ATP synthesis upon downregulation of NDH-1 genes. Interestingly, NDH-1 proteins interact with ribosomal proteins RpsC and RpsQ, and DNA polymerase DnaE in the subnetwork of functional interactions. The succession of protein connectivity in the subnetwork therefore suggests that DosR possibly communicates latency signals to the respiratory chain through Rv0082, resulting in the shutdown of respiration mediated by NDH-1 followed by growth suppression. Since NAD^+^ pool is essential for TCA cycle and other biosynthetic pathways, it appears that the downregulation of NDH-1 respiration is critical in the early stages of latency, which might lead to the arrest of cell growth and division subsequently. Interestingly, the mutants of NADH dehydrogenase I of *Escherichia coli* show competitive disadvantage in the mixed stationary phase cultures [44]. The inability of these mutants to adapt to the stationary phase might arise due to their inefficiency in transmitting signals downstream to slowdown cellular growth processes. Thus, protein interactions and gene regulatory information support the complex regulatory hierarchy of the genes involved in latency.

**Figure 7:**
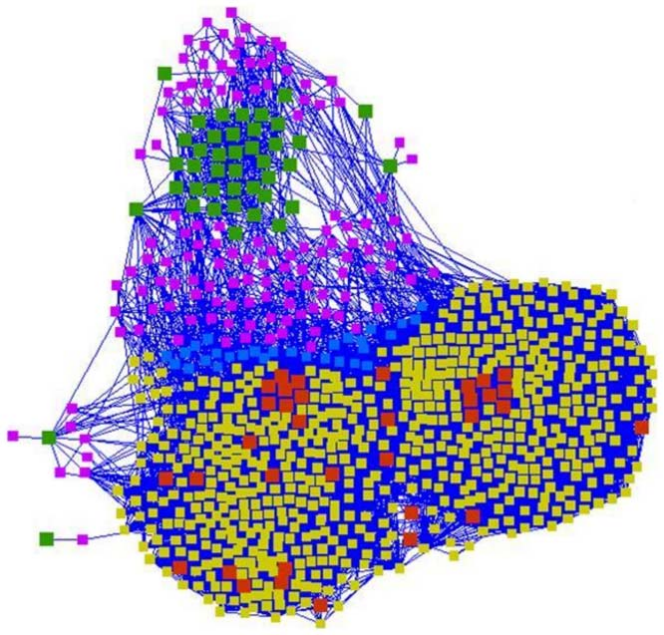
Schematic representation of the up and down-regulated modules. Node color represents the class as follows: Green – Upregulated proteins; Red – Downregulated proteins; Purple – First neighbors of the upregulated proteins; Yellow – First neighbors of the downregulated proteins; Blue – Proteins interacting with both upregulated and downregulated proteins.

In order to further address the complexity of the cross-talk between the up and the downregulated genes in the network, we expanded the network of the 84 genes to construct a “Dormancy Core” comprised of directly interacting proteins of the up and the downregulated genes (Table S8). The “Dormancy Core” was divided into up or downregulated modules depending on the association of the proteins with the up or the downregulated genes. The number of nodes in the upregulated and downregulated modules was 172 and 632 respectively. There are 29 proteins which are common to both these modules (Figure 7). These 29 proteins might participate in direct signalling between the up and the down regulated modules.

Examination of the topological properties of this subnetwork interestingly showed that the nodes in the downregulated module possess higher degree compared to the up-regulated module (Figure S4(A)). As the downregulated module is enriched by proteins of cell growth and division, it is not surprising that these nodes possess higher degree since high degree nodes are more likely to perform essential growth functions [45]. Also, the downregulated module nodes are closer to other proteins in the network as revealed by their closeness centrality compared to the up-regulated module (Figure S4(B)). This suggests their important role in mediating the information flow in the network (Figure S4(C)). However, there is no apparent difference in the clustering coefficients of these two modules (Figure S4(D)). Thus, the known centrality characteristics are able to distinguish between the upregulated and downregulated modules in the network.

The pathway mapping of up and downregulated modules (Figure S5) indicates that the genes from Information pathways are down-regulated during the dormant phase of *M. tuberculosis*. The examples are replication proteins such as DnaA, DnaB, DnaN and GyrA, translation initiation factors such InfA and InfB, proteins of the ribosomal complex, repair and recombination proteins. Interestingly, the subunits of ATP synthase belong to the downregulated module, suggesting an important role of electron transport during dormancy. The down-regulated module also contains Fad proteins which are involved in the degradation of fatty acids, NADH dehydrogenase subunits, proteins involved in polyketides and non-ribosomal peptide synthesis, and cell envelop proteins from the families Lpr and Mur. The up-regulated module, on the other hand, includes the master regulator of dormancy DosR, DosS which functions coordinately with DosR, nitrate reductases NarG, NarJ and NarX, chaperones such as HspX and HtpG, polyketide synthetases such as MbtB and MbtC. Thus, the analysis of the module composition suggests possible pathways that are activated and repressed during the dormant phase of *M. tuberculosis*.

### Shortest Paths Analysis in the Dormancy Module

An interesting aspect of dormancy is the communication between differentially regulated pathways [46], [47]. We sought to identify the possible communication route between the up and downregulated nodes in the dormancy module. All the possible shortest paths were traced from the 50 upregulated nodes to the 34 downregulated nodes and the most probable shortest paths were derived (Methods). The intermediates in the most probable paths were then ranked based on their frequency of occurrence and the top 25% of the intermediates were selected (Table S9). These proteins, we propose, might be involved in transmitting dormancy signals from the upregulated core proteins involved in dormancy, to the downregulated growth module. Below are the few examples we discuss for their possible role in modulating dormancy.

**Figure 8:**
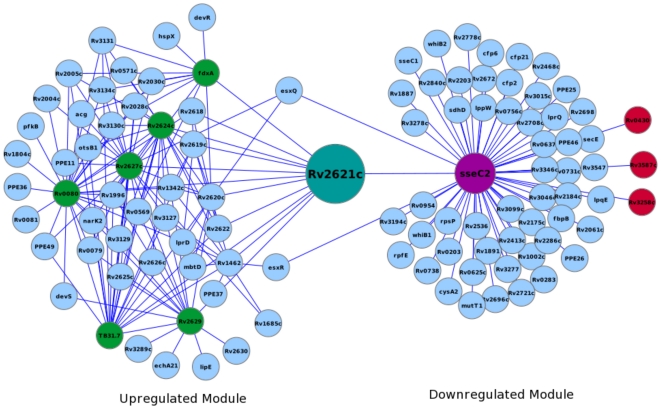
A sub-network depicting the connectivity mediated by proteins Rv2621c and SseC2. Both these proteins occur most frequently in the paths between up and downregulated genes. The upregulated proteins associating with Rv2621c are colored in green and the downregulated proteins associating with SseC2 are colored in red.

Some of the well known dormancy related proteins such as DosR, DosS and HspX occur frequently in the most probable paths calculated. DosR and DosS are the two-component regulatory proteins that have been shown previously to activate a number of proteins in response to the onset of dormancy [32]. HspX, a chaperone, is one such protein from the *dosR* regulon that shows significant induction during dormancy [48]. Another protein coded by *Rv2621c* is a hypothetical transcriptional regulatory protein which interacts with six of the 50 up-regulated nodes. It connects the down-regulated module through SseC2 which is a conserved hypothetical protein thought to be involved in sulphur metabolism. Both these proteins fall frequently in the most probable paths calculated. Figure 8 is a graphical presentation of the interactions of these two proteins. Intriguingly, the interacting partners of SseC2 include WhiB1 and WhiB2, which are transcription factors known to be involved in septation and cell division [49]. They require (4Fe-4S) for the catalytic activity and can function as protein disulfide reductases. The *whiB* homologue of *C. glutamicum* is critical for survival after oxidative stress [50]. Another interacting partner of SseC2 is Rv2175c, which is a transcription factor and a substrate of the PknL Ser/Thr kinase. The vicinity of *Rv2175c* to the *dcw* (division cell wall) cluster to which *pknL* belongs [51] suggests its possible role in regulating cell growth and division. SseC2 also associates with antigen 85B (FbpB) which is a mycolyl transferase involved in cell wall biosynthesis [52]. The cutinase precursor Cfp21, which promotes mycobacterial survival and virulence [53], is another interactor of SseC2. Other secreted proteins such as Cfp21, Cfp2, Rv3194c and Rv2672 also show interaction with SseC2. Thus, the cascade laid by Rv2621c and SseC2 in connecting essential proteins of dormancy to the cell division and growth proteins of the downregulated module appears to be important during switch from/to dormancy.

Another interesting protein of a two-component system is Rv1626, the crystal structure of which suggests its possible role in transcriptional antitermination [54]. In our functional linkage network it interacts with PknA, a Ser/Thr protein kinase and NarK2, a nitrate/nitrite reductase. Both these proteins are members of the up-regulated module. Interestingly, Rv1626 interacts with several down-regulated module proteins, some of which are adenylate kinase Adk, tryptophan synthase TrpA, ribosomal proteins RpsA and RplT, and a two-component transcriptional regulator MtrA.

Another example of an important protein in the most probable paths is EsxR, a secreted ESAT-6 like protein. Interestingly, one of the proteins it connects in the down-regulated module is RpfA, which is a resuscitation promoting factor required for resuscitation from dormant state [55]. Notably, Rpfs have been shown to promote growth in *Micrococcus luteus* and are important for virulence [56]. Thus, the connectivity mediated by EsxR and RfpA between up and downregulated modules appears to be important. The subunits of ATP synthase AtpH, AtpG and AtpC also appear in the most frequent paths. Notably, an inhibitor diarylquinoline targets the proton pump of ATP synthase in *M. tuberculosis* [57]. We may therefore hypothesize that an alteration in the mode of respiration possibly serves as a signal for growth and it is logical to assume that the proteins of ATP synthase communicate such signals.

### Boolean model simulation

In order to determine the main controllers of the dormancy network, a Boolean model simulation was performed using dormancy module. The transcription factors in the dormancy module were sequentially made active at input, individually as well as in various combinations, and the model simulated.

In Boolean terms, a biologically viable steady state of the dormancy model would have the identified upregulated genes in the ON or active state. Likewise, state of the genes identified as down-regulated during dormancy would be OFF or inactive. As mentioned in the methods section, the model included 26 (out of the 50) identified up-regulated genes and 13 (out of 34) down regulated genes. We then set out to determine which of the transcription factors that are known to play important roles in the dormancy network, give rise to a steady state with the required set of genes being active/inactive.

Setting individual transcription factors ON at input resulted in the system having a steady state with all the genes in the inactive or OFF state. However, when three regulatory elements, namely, *Rv0081*, *Rv3132c* (DosS) and *Rv3133c* (DosR), were all set to ON at input, we arrive at a logical steady state, with a number of genes in the active or ON state. Out of these genes, 19 are found to belong to the set of 26 dormancy genes. The simulations therefore confirm that *DosR* and *DosS*, along with *Rv0081* are important regulators in the model.

The cAMP receptor protein (CRP) Rv3676 is known to regulate the expression of a few down-regulated genes. In order to determine its effect on the dormancy system, we added CRP along with the earlier input set (*Rv3132c*, *Rv3133c* and *Rv0081*). The simulation gave rise to a logical attractor cycle, with two states. An attractor cycle with two states implies that the system switches between the two states in an alternate manner. In the first state, while 24 out of the 26 up-regulated genes are active, all 13 down-regulated genes are inactive. However, the second attractor state had only 11 of the down-regulated genes inactive, with *Rv2986c (hupB)* and *Rv1158c* being active. In this state only 19 of the up-regulated genes were active. Hence when the CRP transcription regulator (*Rv3676*) is active at input, two of the downregulated genes, namely, *Rv1158c* and *Rv2986c*, toggle between active and inactive states (Table S10).

In the dormancy module, sigma factor C (Rv2069) regulates the genes *hspX*, *pfkB*, *Rv2030* and *Rv2028c*, which are upregulated during dormancy. In order to test the regulatory effect of SigC in achieving steady state during latency, we included SigC in the set of active input genes along with *Rv3132c*, *Rv3133c*, *Rv0081* and *Rv3676*. The simulation with such an input state results in a two-state attractor cycle similar what is observed by activating *Rv3132c*, *Rv3133c*, *Rv0081* and *Rv367*. Therefore, it appears that SigC may not be a key regulator of *M. tuberculosis* latency. Our Boolean modeling with dormancy module hence reveals key regulators namely Rv3132c, Rv3133c, Rv0081 and Rv3676 for activating and maintaining *M. tuberculosis* latency.

## Discussion

One of the enigmatic features of tuberculosis is that only about 5–10% of the infected individuals develop active tuberculosis [58]. In rest of the cases, *M. tuberculosis* persists in a dormant or a non-replicative state in human tissues for a prolonged time with a potential to resume growth when conditions favor [59]. Understanding the persistent stage of *M. tuberculosis* has proved challenging and has profound implications in containing the disease as the current anti-tuberculosis drugs target only the cells that are actively growing [59]. Moreover, the micro-environment of the granulomas, where latent *M. tuberculosis* resides, is impermeable to the drugs. Inevitably, the systems level understanding of persistence to describe key players in this process, and their association with other proteins is not understood in great detail.

There are 84 differentially regulated genes which are common among many gene expression studies of various dormancy models of *M. tuberculosis*. Our analysis interestingly reveals that these 84 genes are coordinately regulated not only under dormancy-like conditions, but rather form a regulon-like structure. Among these, the 34 downregulated genes show high evolutionary conservation. We argue that the evolutionary conservation of these genes is due to their participation in basic cellular processes. In contrast, the dormancy signal in *M. tuberculosis* appears to be unique, as evidenced by far less conservation of the upregulated genes. It might therefore appear that different bacteria have adopted different mechanisms of entering dormancy, leading eventually to shutting down of the highly conserved basic metabolic processes.

Having identified two distinct clusters of genes, 50 upregulated and 34 downregulated, it is important to understand how these might be coordinately regulated. We used Boolean modeling to examine such transition to dormant phase in *M. tuberculosis* through the 84 differentially regulated genes. In Boolean modeling, an attractor state is the terminal vertex of the state transition graph, i.e. the state to which the system will converge given a certain input state. The attractor state can either be a single steady state, or a set of states through which the system cycles [60]. The latency model developed in this study attempts to understand the key regulators, which when turned on, results in an attractor state that mimics the latent phase of the pathogen. The latent state which is derived upon the activation of certain transcription factors is determined by comparing the states of the genes in the model with prior knowledge of their upregulation or repression during latency. Boolean modeling is therefore an attractive approach to address coordinated regulation among the 84 genes.

We observe that the model converges to an attractor cycle in which about 92% of the upregulated genes remain active when four of the transcription factors in the model namely, DosS (Rv3132c), DosR (Rv3133c), Rv0081 and CRP (Rv3676) are activated at the input. This suggests that these transcription factors are required to be expressed in order to maintain other members of the dormancy network in an active state. Several experimental studies have indicated involvement of a number of transcription factors in the regulation of initiation of the dormancy state [59], [61]. Therefore, the above mentioned transcription factors are not necessarily the only regulators of these processes. However, what the model indicates is that they are the minimum set of transcription factors required to obtain a system that exists in a dormancy-like state. While other factors are most likely involved in establishing the latent condition and adapting to external disturbances from the host macrophage, these four regulatory proteins appear to be the core set of regulators for initiating and maintaining signals for latency.

Two of the regulators predicted to be important for *M. tuberculosis* latency by Boolean modeling are DosR and DosS. Together they form a two-component regulatory system in which DosS is the sensory kinase and DosR is the corresponding response regulator [41]. These regulators have earlier been implicated as key mediators of latency in several experimental studies [59]. For example, rapid induction of *dosR* and *dosS* is observed upon reduced oxygen tension [31]. Similarly, targeted disruption of *dosR* revealed that most of the genes that are induced by hypoxia are regulated by DosR [32]. In addition, DosR and DosS are the members of the ‘dormancy regulon’ identified upon NO treatment [36]. Furthermore, significant load of bacilli and hypervirulence was observed in a SCID mouse model which was infected with *M. tuberculosis* with *dosR* deletion [62]. Activation of DosR is mediated by DosS, which has been established as a redox sensor with O_2_, NO and CO as modulatory ligands [43]. Hence, the two-component system DosR-DosS appears to play a major role in initiating and maintaining latency. Our results using Boolean modeling and network analysis reinforce the importance of these two proteins in establishing latency in *M. tuberculosis*.

Another regulatory protein predicted by Boolean modeling to be important for latency is Rv3676, a cAMP receptor family protein (CRP), which is a global regulator of number of pathways. Computational identification of possible regulatory sites for CRP has earlier revealed a number of genes, some of which are implicated in starvation and hypoxic conditions [63]. Therefore, the association of Rv3676 in latency appears significant. Although there are no reports on the direct involvement of this protein in latency, it will be interesting to study the regulatory role of Rv3676 in this context. Thus, we hypothesize that Rv3676, by virtue of being a global regulator, might influence cross talk among the genes involved in latency.

The fourth important transcriptional regulator suggested by Boolean modeling in *M. tuberculosis* latency is Rv0081, which is a regulatory protein from the ArsR/SmtB family [64]. *Rv0081* is the first gene in the operonic locus *Rv0081–Rv0088*, which codes for the components of formate dehydrogenase complex. *Rv0081* has been observed to be upregulated in multiple latency models, and is also shown to be regulated by DosR in the transcription regulatory network of *M. tuberculosis* [12]. Interestingly, the functional interaction network predicted by us in this study places Rv0082 at the intersection of upregulated and downregulated gene clusters. By being on an operon it might be assumed that Rv0081 controls the transcription of *Rv0082*. Thus, the predicted functional interaction network and Boolean modeling together suggest important roles of Rv0081 and Rv0082 in communicating latency signals between modules of upregulated and downregulated genes in *M. tuberculosis* latency.

The network based approach supplemented with Boolean modeling in elucidating crosstalk between the upregulated and downregulated genes leads us to propose a fascinating hypothesis of the latency process. We observe that DosR plays an important regulatory role in the dormancy switch. The role of DosR in dormancy is well documented in literature [31], [42]. The dormancy signals sensed by two-component sensor kinases, DosS, DosT and possibly by Rv0845 are transmitted to DosR which is a cognate response regulator. In the downstream, the signal is relayed through Rv0081 to the respiratory chain mediated by the Rv0082. The information flow from Rv0082 then triggers slowing down of ATP synthesis, leading eventually to significant slowing down of replication, transcription and translation processes. Thus, through an intricate communication signal, the basic cellular processes such as cell division and growth are shut down. Some of the hypotheses proposed in our work will obviously need to be tested experimentally.

## Methods

### Positive and Negative Interaction Pairs

The known interacting protein pairs were obtained by a combination of text mining methods and bi-directional blast against high confident protein interactions for *E. coli* listed in EcoCyc database [65]. Those obtained by text mining were retrieved from literature by the use of natural language processing methods (Goyal and Mande, unpublished results). Bi-directional BLAST was carried out for each pair listed in the EcoCyc database against the *M. tuberculosis* H37Rv genome sequence. Only those pairs were further considered which showed a score better than e^−10^ for both the proteins.

The hypothesized non-interacting data set was obtained by the methods described in [25]. Briefly, the protein pairs which are not colocalised in the same subcellular compartment were considered to be non-interacting. The protein localization was predicted using the SIGCLEAVE tool available at http://mobyle.pasteur.fr/cgi-bin/Mobyle Portal. The top scoring 809 proteins with predicted secretary signal sequence within the first 50 residues of the N-terminus were considered to be extracellular. There are 161 proteins which do not possess any known signal sequence along their entire length and were considered to be cytoplasmic. Such negative interacting protein pairs were generated by randomly pairing the predicted cytoplasmic and extracellular proteins.

### Selection of the Genomes

The sequences of 763 bacterial genomes were downloaded from NCBI ftp site (ftp://ncbi.nih.gov/genomes/Bacteria). In the initial filter, bacteria with linear or multiple genomes were removed. This resulted in a set of 669 genomes for further analysis. Homologous genes of all the known open reading frames of *M. tuberculosis* were searched against these 669 genomes using BLASTp with e-value cutoff of e^−04^. For the species with complete genome sequences of more than one strain, the one which shared maximum number of ORFs with *M. tuberculosis* was chosen. This resulted in a list of 481 genomes for further consideration (Table S11).

### Prediction Features

#### Phylogenetic Profile

BLAST with e-value cutoff of e^−04^ was used to obtain bit scores for the ORFs of *M. tuberculosis* against 481 selected genomes. The resulting profile was doubly normalized as in [25]. Pearson Correlation Coefficient (PCC) was calculated for each gene pair and used as a feature for training the SVM.

#### Intergenic Distance

For using minimum gene distance as a training feature, top 100 organisms sharing maximum number of ORFs with *M. tuberculosis* were considered. For each genome, distances (in base pairs) of transcriptional start sites between all the gene pairs were calculated in both clockwise and anticlockwise directions and the minimum of these was normalized by the total genome length. A similar profile was constructed for *M. tuberculosis* genome as well. For each gene pair of *M. tuberculosis*, the minimum distance in its genome and its orthologs in other genomes was considered as a feature vector.

#### Frequency of co-occurrence in the predicted operons

Operon predictions for 267 organisms were obtained from [25]. The frequency of co-occurrence of protein pairs as operonic across all the genomes was calculated.

#### Expression Correlations

Gene expression data for *M. tuberculosis* was downloaded from NCBI-Geo [6]. The expression values for the multiple trails were normalized. The conditions which had expression variance of more than 5 were not considered for the analysis. The expression ratio in 154 growth conditions for each gene was compiled and Pearson Correlation Coefficient was derived for each gene pair. The list of selected conditions is detailed in Table S12.

### Protein Interactions Prediction

Support Vector Machine (SVM) tool LibSVM [66] was used for the prediction of genome-wide functional linkages. All possible gene pairs in *M. tuberculosis* carried the feature vector labels, namely correlation coefficient of the phylogenetic profile, minimum intergenic distance, frequency of co-occurrence in predicted operons and expression correlations. The machine was trained on positive and negative data sets using these data features. Since the number of expected interacting pairs was likely to be much lower than the non-interacting pairs, the ratio of negative interacting pairs and the positive interacting pairs was increased for each trial. Each test included five fold cross validation followed by calculations of sensitivity and specificity. The interactions were predicted with each of the model files obtained and the final network was selected based on sensitivity, specificity and the accuracy of prediction. The final predictions are available on our web server (http://www.nccs.res.in/MtbPPI/).

### Network Analysis

Different topological parameters of the network that included degree exponent, clustering coefficient and diameter were calculated according to [67]. Centrality measures were calculated as in [28]. Clusters in the network were detected using the Infomap tool [68]. Functional annotations of *M. tuberculosis* proteins were derived from KEGG [69], TubercuList (http://genolist.pasteur.fr/TubercuList/) and Sanger (http://www.sanger.ac.uk/) databases. Clustering coefficient or degree for a pathway is considered to be high if the average clustering coefficient or the average degree for the proteins in the pathway was more than the average of all the proteins. The shortest paths were calculated using Dijkstra's algorithm [70]. Since there can be more than one shortest path for a pair of nodes, most probable paths were derived by considering most frequently occurring proteins in the shortest paths at each position.

Combined subnetwork was constructed by merging protein functional linkages and gene regulatory interactions [12]. If the interaction for two proteins is represented as both protein functional linkage as well as gene regulatory interaction, the latter was considered for the analysis.

### Boolean modeling

Boolean models consist of components or nodes with logical rules or transfer functions governing their interactions. The state of each node in a typical boolean network is usually **o**nly a logical value, namely ‘ON’/‘1’ or ‘OFF’/‘0’. In the case of a gene regulatory network, the ‘ON’ state can be considered to represent a gene being activated or expressed and the ‘OFF’ state as a gene being inactivated or unexpressed. The state of a node at time *t+1* is derived from states of all the nodes which interact with it at time *t*, by means of a logical function or rule [71]. The states are updated by means various updating schemes, the simplest one being synchronous updating where all the nodes are updated simultaneously at a given time point. This is in contrast to asynchronous updating, where a random node is selected at each time step and updated. The model dynamics comprise of various states the different components pass through to converge at an attractor state (fixed point or cycle). The attractor states of a model correspond to various biological states of the system under study.

### Model of *Mycobacterium tuberculosis* dormancy network

A large-scale boolean network model was constructed based on a subset of genes identified as being differentially regulated and involved during the early dormancy state in *M. tuberculosis*. A number of criteria were considered when selecting components for the Boolean model. To begin with, genes annotated as transcription factors or regulatory elements which were found to be expressed in a minimum of 5 experimental latency conditions were identified. The expression correlation values for these transcription factors and their corresponding interactions were also obtained. To this list, the regulatory interactions determined in [12] were also added.

This list of genes was then narrowed down by discarding genes which were activated or inhibited by only a single transcription factor, with the interaction being insignificant. An interaction was considered to be insignificant if the expression correlation between the two members was between −0.3 and +0.3, and significant otherwise. Furthermore, if a given gene was regulated by a single transcription factor and that transcription factor itself had no regulatory elements acting on it, then that gene was removed as well.

The resulting subset consisted of genes or nodes, and 543 interactions or arcs, with 46 found to be annotated as regulatory elements such as transcription factors. Among the 304 genes, 26 belonged to the group of 50 genes identified as being upregulated during dormancy (Table S7). Similarly, 13 out of the 34 genes identified as being downregulated were present in the model.

The network was built by obtaining the interactions identified for each of the 304 genes from the protein functional linkages. As we had selected only transcription factors and the genes the regulated, the interactions were all directed, i.e. those originating from a transcription factor or regulatory element. We then derived the logical rule for each gene using the calculated expression correlation values for these interactions, and employing the following criteria:

For a given gene *X*,

- If a set of transcription factors were positively correlated with X, then the correlation between them was detemernied. If the correlation was significant, then all of them were considered to be required for the activation or expression of X. A logical AND was used in this case.
- If a set of transcription factors were positively and signifcantly correlated with X but insignificantly correlated with each other, then any one of them was sufficient for the activation of X. A logical OR was used in this case.
- If a set of transcription factors were negatively correlated with *X* and positively correlated with each other, then all of them were considered to be required for the inhibition or inactivation of *X*. A logical NOT AND was used in this case.
- If a set of transcription factors were negatively correlated with X and insiginificantly correlated with each other, then any one of them was considered to be sufficient for the inactivation of X. A logical NOT OR was used in this case.

The detailed list of logical rules is given in Table S13.

In our model, we do not differentiate between the expression and regulation of a gene but represent them in an equivalent manner. This biological simplification is necessary given that only expression correlation values were available to us. Furthermore, while significant expression correlation between the a gene and transcription factor might arise due to various forms of activation or regulation, we feel safe to represent it as direct in this case, given that the genes were obtained from the set determined to form a regulon-like structure. The model was simulated by setting as ON (i.e ‘1’) the individual transcription factors and updating all the genes in a synchronous manner. The simulations were done with the transcription factors being set as ON simultaneously, as well as one by one.

The boolean model was developed and simulated using the logical modelling, analysis and simulation software *GINsim* [72].

## Supporting Information

Figure S1 Plot of ROC depicting the performance of SVM.
(TIF)

Figure S2 Plot denoting enrichment of Up and Downregulated dormancy genes to different conservation bins. Upregulated genes are less conserved than downregulated genes.
(TIF)

Figure S3 Expression correlation between genes coding for NDH-1 subunits and the genes coding for ATP synthase subunits. The plot indicates that NDH-1 genes and ATP synthase genes are correlated in their expression.
(TIF)

Figure S4 Differential Network Properties of the Modules of up and downregulated genes. Proteins in the downregulated module show high degree centrality (Figure S4(A), P<2.2e^−16^), high closeness centrality (Figure S4(B), P<2.2e^−16^) and high betweenness centrality (Figure S4(C), P<3.9e^−09^) compared to the proteins in the upregulated module. However, there is no apparent difference in their clustering coefficients (Figure S4(D), P<0.44).
(TIF)

Figure S5 TubercuList pathway map of the proteins belonging to up and down-regulated modules. Genes belonging to Information pathway are significantly downregulated during dormancy.
(TIF)

Table S1 Predicted Protein functional linkages of *M. tuberculosis*.
(TXT)

Table S2 Comparison of predicted interactions with other available interactions. There is about 30% overlap between predicted interactions and previous reports.
(DOC)

Table S3 List of high centrality proteins in the network.
(TXT)

Table S4 List of communities identified in the interaction map using Infomap community detection tool.
(TXT)

Table S5 List of publications related to dormancy models used in this study and the number of up and down-regulated genes in each model.
(DOC)

Table S6 Comparison of different dormancy models in terms of number of genes up and downregulated in each.
(DOC)

Table S7 List of up and downregulated genes during dormancy and their functions.
(TXT)

Table S8 List of proteins in the Dormancy Core.
(TXT)

Table S9 List of proteins possibly mediating the dormancy signals between up and down-regulated modules.
(TXT)

Table S10 Genes which are active at the logical steady state reached by the system when three regulatory elements are ON at input (*Rv0081*, *Rv3132c* and *Rv3133c*) and when four regulatory elements are ON at input (*Rv0081*, *Rv1343c*, *Rv3132c* and *Rv3133c*).
(DOC)

Table S11 List of selected genomes for constructing phylogenetic profile.
(TXT)

Table S12 List of microarray conditions used for calculating expression correlations.
(TXT)

Table S13 Logical rules derived from expression data for each of the nodes in the simulated model.
(DOC)
