# Supplementary figures and images for: Understanding Communication Signals during Mycobacterial Latency through Predicted Genome-Wide Protein Interactions and Boolean Modeling
Source: PLoS One. 2012 Mar 20;7(3):e33893. doi: 10.1371/journal.pone.0033893 (PMC3309013; doi:10.1371/journal.pone.0033893)

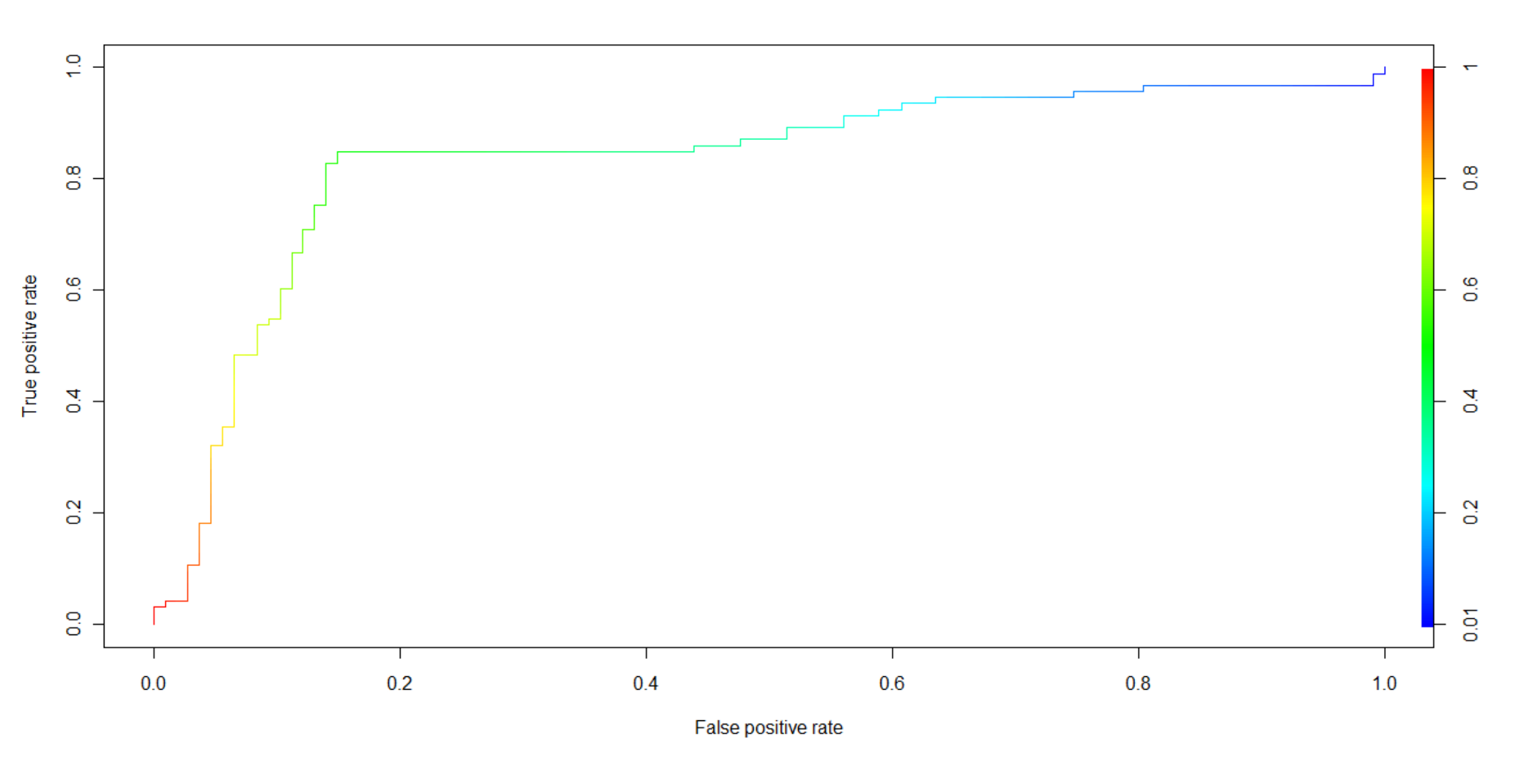

Supplement: Figure S1 — Plot of ROC depicting the performance of SVM. (TIF) [file pone.0033893.s001.tif]

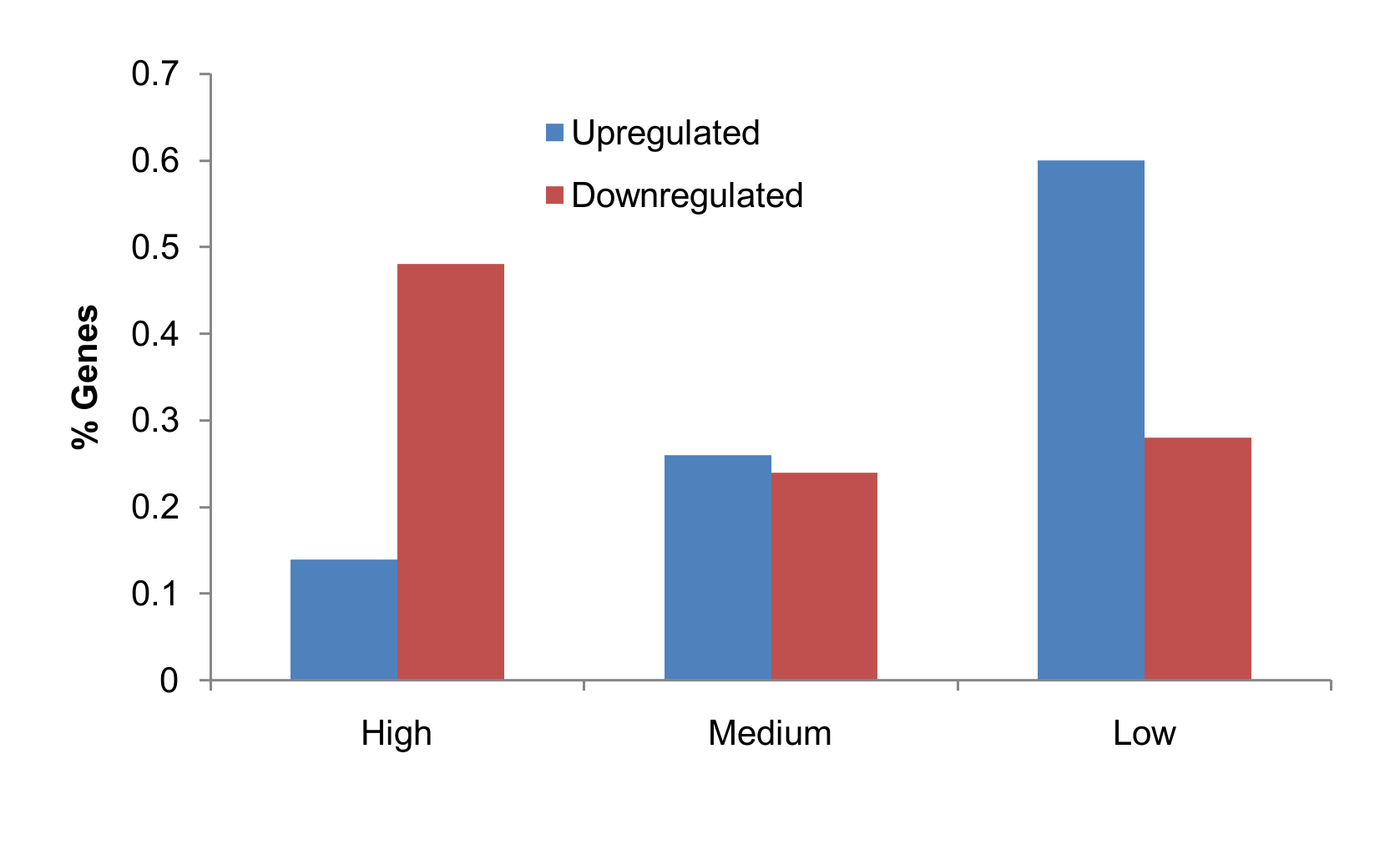

Supplement: Figure S2 — Plot denoting enrichment of Up and Downregulated dormancy genes to different conservation bins. Upregulated genes are less conserved than downregulated genes. (TIF) [file pone.0033893.s002.tif]

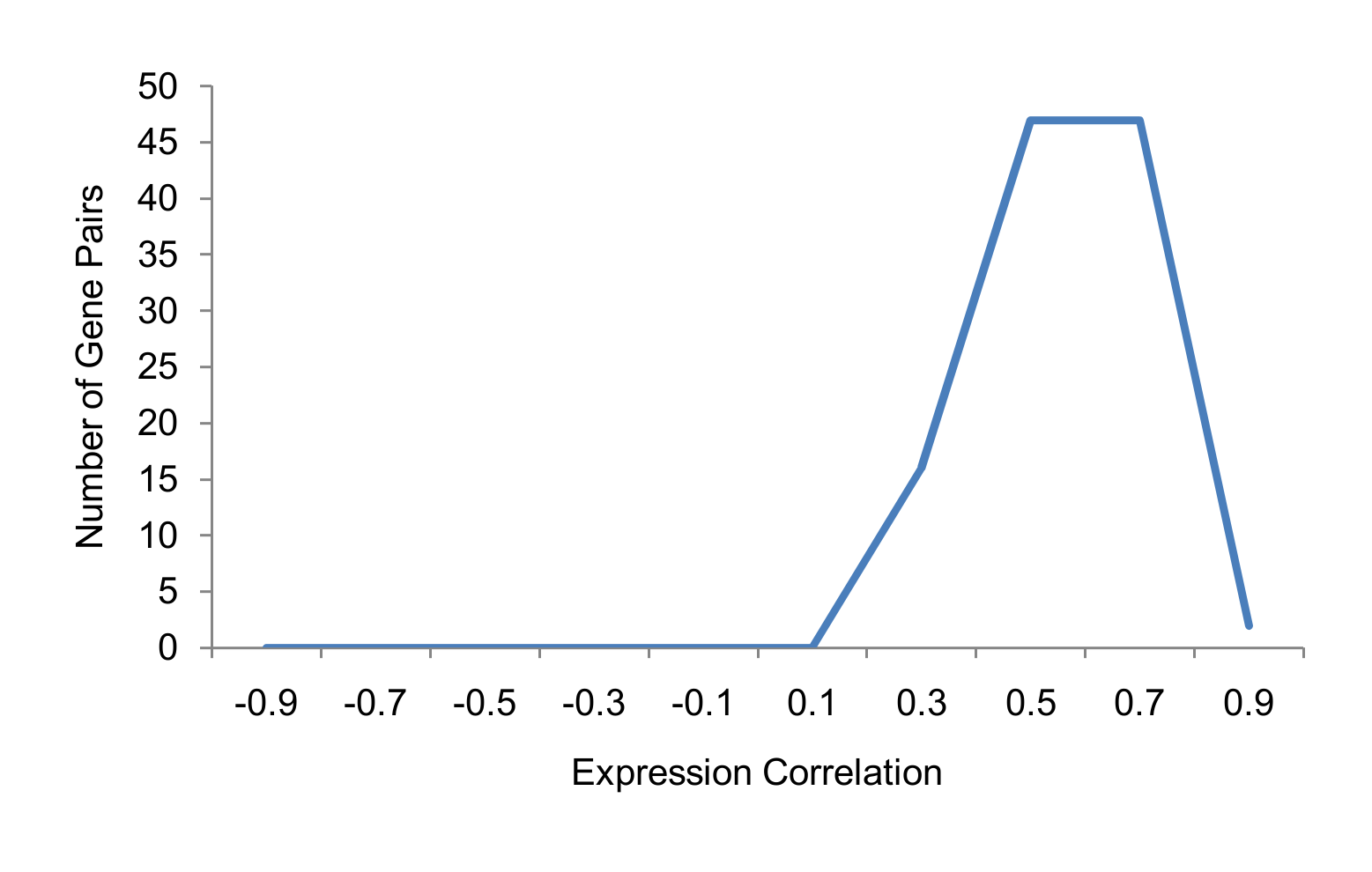

Supplement: Figure S3 — Expression correlation between genes coding for NDH-1 subunits and the genes coding for ATP synthase subunits. The plot indicates that NDH-1 genes and ATP synthase genes are correlated in their expression. (TIF) [file pone.0033893.s003.tif]

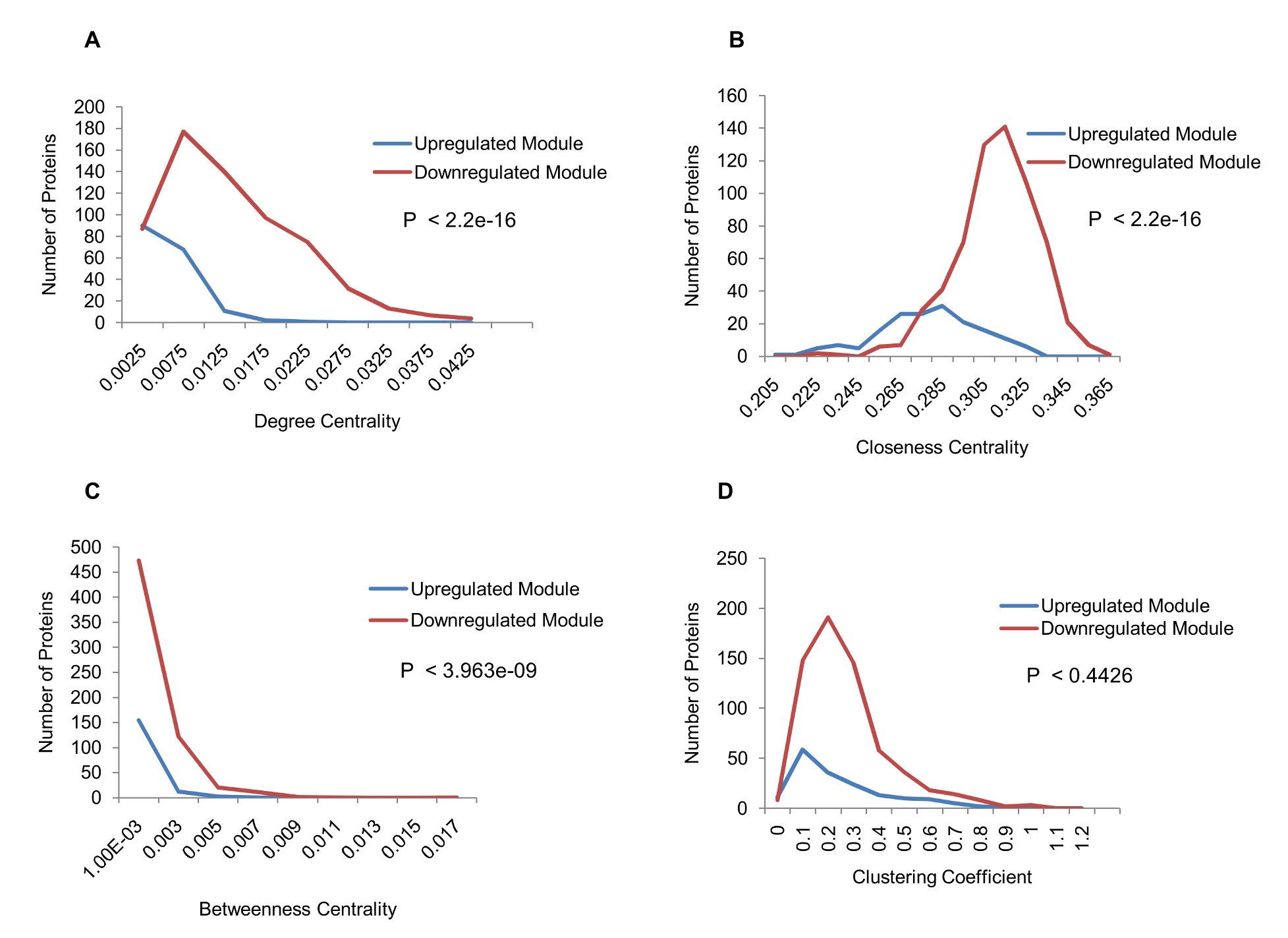

Supplement: Figure S4 — Differential Network Properties of the Modules of up and downregulated genes. Proteins in the downregulated module show high degree centrality (Figure S4(A), P<2.2e−16), high closeness centrality (Figure S4(B), P<2.2e−16) and high betweenness centrality (Figure S4(C), P<3.9e−09) compared to the proteins in the upregulated module. However, there is no apparent difference in their clustering coefficients (Figure S4(D), P<0.44). (TIF) [file pone.0033893.s004.tif]

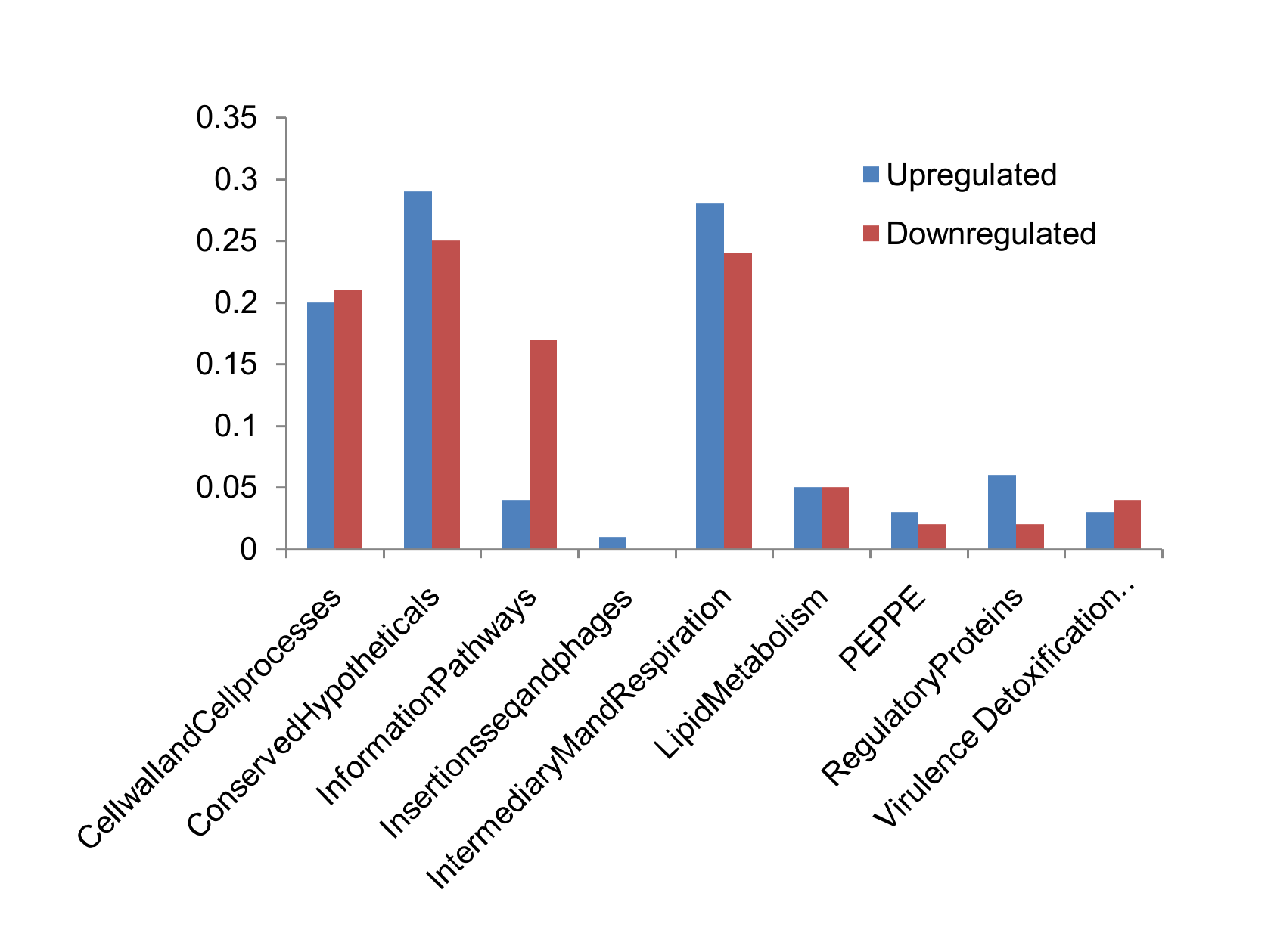

Supplement: Figure S5 — TubercuList pathway map of the proteins belonging to up and down-regulated modules. Genes belonging to Information pathway are significantly downregulated during dormancy. (TIF) [file pone.0033893.s005.tif]
