# Supplementary material for: Understanding Communication Signals during Mycobacterial Latency through Predicted Genome-Wide Protein Interactions and Boolean Modeling
Source: PLoS One. 2012 Mar 20;7(3):e33893. doi: 10.1371/journal.pone.0033893 (PMC3309013; doi:10.1371/journal.pone.0033893)
Supplement: Table S2 — Comparison of predicted interactions with other available interactions. There is about 30% overlap between predicted interactions and previous reports. (DOC) [file pone.0033893.s007.doc]

**Table S2:** Comparison of predicted interactions with other available interactions. There is about 30% overlap between predicted interactions and the previous reports.

| **Network** | **Interactions** | **Nodes** | **Common Interactions** |
| --- | --- | --- | --- |
| **STRING database (High-confidence Interactions)** | 6403 | 1653 | 1964 |
| **Strong et al (Nucleic Acids Research, 2003)** | 4886 | 1958 | 1485 |
| **Cui et al (BMC Genomics, 2009)** | 6091 | 793 | 459 |
