# Supplementary material for: Understanding Communication Signals during Mycobacterial Latency through Predicted Genome-Wide Protein Interactions and Boolean Modeling
Source: PLoS One. 2012 Mar 20;7(3):e33893. doi: 10.1371/journal.pone.0033893 (PMC3309013; doi:10.1371/journal.pone.0033893)
Supplement: Table S5 — List of publications related to dormancy models used in this study and the number of up and down-regulated genes in each model. (DOC) [file pone.0033893.s010.doc]

**Table S5:** List of publications related to dormancy models used in this study and the number of up and down-regulated genes in each model.

| **Experimental Condition** | **Up-regulated Genes** | **Down-regulated Genes** |
| --- | --- | --- |
| **O_2_ Depletion** | | |
| Park et al, Mol Microbiol, 2003 | 161 | 71 |
| Sherman et al, PNAS, 2001 | 135 | 79 |
| Muttucumaru et al, Tuberculosis, 2004 | 358 | 381 |
| Bacon et al, Tuberculosis, 2004 | 144 | 55 |
| Voskuil et al, Tuberculosis, 2004  i) NRP Day 6  II) NRP Day 8 | 94  116 | 279  350 |
| **Stationary Phase** | | |
| Voskuil et al, Tuberculosis, 2004  i) Stationary Phase Day 6  ii) Stationary Phase Day 8 | 15  39 | 139  222 |
| **NO Model** | | |
| Voskuil et al, J. Exp. Med, 2003 | 223 | 181 |
| **Starvation Model** | | |
| Betts et al, Mol Microbiol, 2002 | 170 | 211 |
| **Murine Model** | | |
| Schnappinger et al, J. Exp, Med, 2003 | 154 | 90 |
| Karakousis et al, J. Exp. Med, 2004 | 253 | 49 |
