# Supplementary material for: Understanding Communication Signals during Mycobacterial Latency through Predicted Genome-Wide Protein Interactions and Boolean Modeling
Source: PLoS One. 2012 Mar 20;7(3):e33893. doi: 10.1371/journal.pone.0033893 (PMC3309013; doi:10.1371/journal.pone.0033893)
Supplement: Table S6 — Comparison of different dormancy models in terms of number of genes up and downregulated in each. (DOC) [file pone.0033893.s011.doc]

**Table S6:** Comparison of different dormancy models in terms of number of genes up and downregulated in each

***Down-regulated Genes***

|  | **Murine Model**  **[127]** | **NO Model**  **[181]** | **O_2_ Model**  **[828]** | **Stationary Phase [277]** | **Starvation Model [211]** |
| --- | --- | --- | --- | --- | --- |
| **Murine Model**  **[127]** | - | **28** | **73** | **22** | **10** |
| **NO Model**  **[181]** | **61** | - | **59** | **37** | **17** |
| **O_2_ Model**  **[828]** | **130** | **108** | - | **83** | **68** |
| **Stationary Phase [277]** | **14** | **12** | **15** | - | **32** |
| **Starvation Model [211]** | **20** | **20** | **30** | **10** | - |

***Up-regulated Genes***
