# Supplementary material for: Understanding Communication Signals during Mycobacterial Latency through Predicted Genome-Wide Protein Interactions and Boolean Modeling
Source: PLoS One. 2012 Mar 20;7(3):e33893. doi: 10.1371/journal.pone.0033893 (PMC3309013; doi:10.1371/journal.pone.0033893)
Supplement: Table S10 — Genes which are active at the logical steady state reached by the system when three regulatory elements are ON at input (Rv0081, Rv3132c and Rv3133c) and when four regulatory elements are ON at input (Rv0081, Rv1343c, Rv3132c and Rv3133c). (DOC) [file pone.0033893.s015.doc]

**Table S10:** Genes from the up-regulated data set which are active at the logical steady state reached by the system when three regulatory elements are ON at input (Rv0081, Rv3132c and Rv3133c) and when four regulatory elements are ON at input (Rv0081, Rv3132c, Rv3133c and Rv3676).

|  | **Input ON: Rv0081, Rv3132c, Rv3133c** | **Stable state** | **Input ON: Rv0081, Rv3132, Rv3133c, Rv3676** | **Attractor cycle state 1** | **Attractor cycle state 2** |
| --- | --- | --- | --- | --- | --- |
| 1. | Rv0079 | 1 | Rv0079 | 1 | 1 |
| 2. | Rv0081 | 1 | Rv0080 | 0 | 1 |
| 3. | Rv0082 | 1 | Rv0081 | 1 | 1 |
| 4. | Rv0569 | 1 | Rv0082 | 1 | 1 |
| 5. | Rv1736c | 1 | Rv0569 | 1 | 1 |
| 6. | Rv1996 | 1 | Rv1592c | 1 | 1 |
| 7. | Rv2005c | 1 | Rv1736c | 1 | 1 |
| 8. | Rv2006 | 1 | Rv1996 | 1 | 1 |
| 9. | Rv2029c | 1 | Rv1997 | 1 | 1 |
| 10. | Rv2030c | 1 | Rv2005c | 1 | 1 |
| 11. | Rv2031c | 1 | Rv2006 | 1 | 1 |
| 12. | Rv2032 | 1 | Rv2028c | 0 | 1 |
| 13. | Rv2629 | 1 | Rv2029c | 1 | 1 |
| 14. | Rv3127 | 1 | Rv2030c | 1 | 1 |
| 15. | Rv3129 | 1 | Rv2031c | 1 | 1 |
| 16. | Rv3130c | 1 | Rv2032 | 1 | 1 |
| 17. | Rv3132c | 1 | Rv2629 | 1 | 1 |
| 18. | Rv3133c | 1 | Rv3127 | 0 | 1 |
| 19. | Rv3134c | 1 | Rv3129 | 1 | 1 |
| 20. |  |  | Rv3130c | 1 | 1 |
| 21. |  |  | Rv3131 | 0 | 1 |
| 22. |  |  | Rv3132c | 1 | 1 |
| 23. |  |  | Rv3133c | 1 | 1 |
| 24. |  |  | Rv3134c | 1 | 1 |
