# Supplementary material for: Understanding Communication Signals during Mycobacterial Latency through Predicted Genome-Wide Protein Interactions and Boolean Modeling
Source: PLoS One. 2012 Mar 20;7(3):e33893. doi: 10.1371/journal.pone.0033893 (PMC3309013; doi:10.1371/journal.pone.0033893)
Supplement: Table S13 — Logical rules derived from expression data for each of the nodes in the simulated model. (DOC) [file pone.0033893.s018.doc]

| **Gene** | **Transcription factors with correlation** | **Correlations between the transcripton factors** | **Logical Rule** |
| --- | --- | --- | --- |
| Rv0001 | Rv1985c (-0.358339) | -0.36 | = Rv1985 |
| Rv0002 | Rv0001 (0.285470) Rv1985c (-0.337699) Rv2711 (-0.387199) Rv3676 (0.151194) | Rv0001 Rv1985c -3.5833910e-001 Rv0001 Rv2711 3.6316095e-002 Rv0001 Rv3676 5.6495882e-001 Rv1985c Rv2711 3.0454957e-001 Rv1985c Rv3676 8.3208446e-003 Rv2711 Rv3676 3.6994229e-002 | = (Rv0001 AND Rv3676) OR (NOT Rv1985 AND NOT Rv2711) |
| Rv0005 | Rv3676 (0.465689) | 0.47 | = Rv3676 |
| Rv0006 | Rv3676 (0.488480) | 0.49 | = Rv3676 |
| Rv0007 | Rv0014c (0.706668) Rv3414c (0.546928) Rv3676 (0.365377) | Rv0014c Rv3414c 5.2640525e-001 Rv0014c Rv3676 4.1556177e-001 Rv3414c Rv3676 4.4032942e-001 | = Rv3676 AND Rv0014 AND Rv3414 |
| Rv0009 | Rv2027c (-0.197395) Rv3133c (-0.074206) Rv3286c (-0.108956) Rv3676 (0.584923) | Rv2027c Rv3133c 1.2017934e-001 Rv2027c Rv3286c 1.9346813e-001 Rv2027c Rv3676 1.3191918e-001 Rv3133c Rv3286c 1.6144858e-001 Rv3133c Rv3676 -4.9377524e-002 Rv3286c Rv3676 1.5929419e-001 | = Rv3676 OR (NOT Rv2027 OR Rv3133c OR Rv3286c) |
| Rv0011c | Rv0020c (0.730113) Rv3286c (0.026429) | Rv0020c Rv3286c -1.0629458e-001 | = Rv0020 OR Rv3286c |
| Rv0014c | Rv0015c (0.493176) Rv0019c (0.056909) Rv0020c (0.473340) | Rv0015c Rv0019c 3.9021494e-001 Rv0015c Rv0020c 3.1193993e-001 Rv0019c Rv0020c 4.2907013e-001 | = Rv0015c AND Rv0019c AND Rv0020c |
| Rv0015c | Rv0014c (0.493176) | 0.49 | = Rv0014c |
| Rv0016c | Rv0014c (0.684129) Rv0015c (0.319757) Rv0019c (0.056174) Rv0020c (0.384893) | Rv0014c Rv0015c 4.9317619e-001 Rv0014c Rv0019c 5.6908563e-002 Rv0014c Rv0020c 4.7334014e-001 Rv0015c Rv0019c 3.9021494e-001 Rv0015c Rv0020c 3.1193993e-001 Rv0019c Rv0020c 4.2907013e-001 | = Rv0014c AND Rv0015c AND Rv0020c AND Rv0019c |
| Rv0019c | Rv0014c (0.056909) Rv0015c (0.390215) Rv3676 (0.265217) | Rv0014c Rv0015c 4.9317619e-001 Rv0014c Rv3676 4.1556177e-001 Rv0015c Rv3676 2.7486313e-001 | = Rv0014c AND Rv0015c AND Rv3676 |
| Rv0020c | Rv0014c (0.473340) Rv0015c (0.311940) Rv0019c (0.429070) | Rv0014c Rv0015c 4.9317619e-001 Rv0014c Rv0019c 5.6908563e-002 Rv0015c Rv0019c 3.9021494e-001 | = Rv0014c AND Rv0015c AND Rv0019c |
| Rv0053 | Rv1379 (0.836969) Rv2720 (-0.116162) | Rv1379 Rv2720 -2.3839590e-001 | = Rv1379 OR NOT Rv2720 |
| Rv0054 | Rv1379 (0.788063) Rv2720 (-0.017133) | Rv1379 Rv2720 -2.3839590e-001 | = Rv1379 OR NOT Rv2720 |
| Rv0055 | Rv2720 (-0.025981) Rv3246c (0.545924) | Rv2720 Rv3246c 2.0148206e-001 | = Rv3246c OR NOT Rv2720 |
| Rv0056 | Rv2720 (-0.071235) Rv3246c (0.605509) | Rv2720 Rv3246c 2.0148206e-001 | = Rv3246c OR NOT Rv2720 |
| Rv0058 | Rv2720 (0.015419) Rv3246c (0.639156) | Rv2720 Rv3246c 2.0148206e-001 | = Rv3246c OR NOT Rv2720 |
| Rv0079 | Rv3132c (0.644837) | 0.64 | = Rv3132c |
| Rv0080 | Rv0081 (0.835423) Rv1343c (0.632432) Rv2621c (0.443405) Rv3132c (0.674844) | Rv0081 Rv1343c 4.6723610e-001 Rv0081 Rv2621c 3.4032824e-001 Rv0081 Rv3132c 7.1179390e-001 Rv1343c Rv2621c 5.3811809e-001 Rv1343c Rv3132c 4.5276273e-001 Rv2621c Rv3132c 1.8737765e-001 | = Rv0081 AND Rv1343 AND (Rv2621c OR RV3132c) |
| Rv0081 | Rv3133c (0.749290) | 0.749290 | = Rv3133c |
| Rv0082 | Rv0081 (0.885363) | 0.885363 | = Rv0081 |
| Rv0083 | Rv0081 (0.552887) | 0.552887 | = Rv0081 |
| Rv0084 | Rv0081 (0.708428) | 0.708428 | = Rv0081 |
| Rv0085 | Rv0081 (0.613093) | 0.613093 | = Rv0081 |
| Rv0086 | Rv0081 (0.572893) | 0.572893 | = Rv0081 |
| Rv0087 | Rv0081 (0.443373) | 0.443373 | = Rv0081 |
| Rv0088 | Rv0081 (0.302703) | 0.3 | = Rv0081 |
| Rv0113 | Rv2711 (-0.404423) | -0.4 | = NOT Rv2711 |
| Rv0114 | Rv2711 (-0.468950) | 0.3 | = NOT Rv2711 |
| Rv0117 | TF |  |  |
| Rv0138 | Rv0020c (0.632078) | 0.63 | = Rv0020c |
| Rv0164 | Rv2175c (0.596404) | 0.6 | = Rv2175c |
| Rv0167 | Rv0494 (-0.426408) Rv3676 (0.292436) | Rv0494 Rv3676 -2.2776261e-003 | = Rv3676 OR NOT Rv0494 |
| Rv0168 | Rv0494 (-0.219746) Rv3676 (0.266068) | Rv0494 Rv3676 -2.2776261e-003 | = Rv3676 OR NOT Rv0494 |
| Rv0169 | Rv0014c (0.717632) Rv0494 (-0.418824) Rv3414c (0.635798) Rv3676 (0.401516) | Rv0014c Rv0494 -2.7834227e-001 Rv0014c Rv3414c 5.2640525e-001 Rv0014c Rv3676 4.1556177e-001 Rv0494 Rv3414c -2.6465222e-001 Rv0494 Rv3676 -2.2776261e-003 Rv3414c Rv3676 4.4032942e-001 | = Rv0014c AND Rv3414c AND Rv3676 OR NOT Rv0494 |
| Rv0170 | Rv0494 (-0.242286) Rv3414c (0.648679) Rv3676 (0.346939) | Rv0494 Rv3414c -2.6465222e-001  Rv0494 Rv3676 -2.2776261e-003  Rv3414c Rv3676 4.4032942e-001 | = Rv3414c AND Rv3676 OR NOT Rv0494 |
| Rv0171 | Rv0494 (-0.142271) Rv3676 (0.430260) | Rv0494 Rv3676 -2.2776261e-003 | = Rv3676 OR NOT Rv0494 |
| Rv0172 | Rv0014c (0.731863) Rv0494 (-0.316306) Rv3414c (0.666679) Rv3676 (0.385760) | Rv0014c Rv0494 -2.7834227e-001 Rv0014c Rv3414c 5.2640525e-001 Rv0014c Rv3676 4.1556177e-001 Rv0494 Rv3414c -2.6465222e-001 Rv0494 Rv3676 -2.2776261e-003 Rv3414c Rv3676 4.4032942e-001 | = Rv0014c AND Rv3414c AND Rv3676 OR NOT Rv0494 |
| Rv0173 | Rv0014c (0.753881) Rv0494 (-0.263804) Rv3414c (0.634777) Rv3676 (0.237926) | Rv0014c Rv0494 -2.7834227e-001 Rv0014c Rv3414c 5.2640525e-001 Rv0014c Rv3676 4.1556177e-001 Rv0494 Rv3414c -2.6465222e-001 Rv0494 Rv3676 -2.2776261e-003 Rv3414c Rv3676 4.4032942e-001 | = Rv0014c AND Rv3414c AND Rv3676 OR NOT Rv0494 |
| Rv0174 | Rv0494 (-0.318303) Rv3414c (0.616173) Rv3676 (0.338003) | Rv0494 Rv3414c -2.6465222e-001 Rv0494 Rv3676 -2.2776261e-003 Rv3414c Rv3676 4.4032942e-001 | = Rv3414c AND Rv3676 OR NOT Rv0494 |
| Rv0175 | Rv0020c (0.645757) Rv0494 (-0.476018) Rv2175c (0.529658) Rv3676 (0.364151) | Rv0020c Rv0494 -3.1271691e-001 Rv0020c Rv2175c 3.4868007e-001 Rv0020c Rv3676 3.1881544e-001 Rv0494 Rv2175c -3.4985986e-001 Rv0494 Rv3676 -2.2776261e-003 Rv2175c Rv3676 2.5027063e-001 | = Rv0020 AND Rv3676 AND Rv2175 OR NOT Rv0494 |
| Rv0177 | Rv0014c (0.716305) Rv0020c (0.628932) Rv0494 (-0.474571) Rv2175c (0.415024) Rv3676 (0.542197) | Rv0014c Rv0020c 4.7334014e-001 Rv0014c Rv0494 -2.7834227e-001 Rv0014c Rv2175c 1.4442559e-001 Rv0014c Rv3676 4.1556177e-001 Rv0020c Rv0494 -3.1271691e-001 Rv0020c Rv2175c 3.4868007e-001 Rv0020c Rv3676 3.1881544e-001 Rv0494 Rv2175c -3.4985986e-001 Rv0494 Rv3676 -2.2776261e-003 Rv2175c Rv3676 2.5027063e-001 | = Rv0020 AND Rv0014 AND Rv3676 AND Rv2175 OR NOT Rv0494 |
| Rv0178 | Rv0014c (0.688323) Rv0020c (0.560124) Rv0494 (-0.361583) Rv2175c (0.482234) Rv3414c (0.645693) Rv3676 (0.549174) | Rv0014c Rv0020c 4.7334014e-001 Rv0014c Rv0494 -2.7834227e-001 Rv0014c Rv2175c 1.4442559e-001 Rv0014c Rv3414c 5.2640525e-001 Rv0014c Rv3676 4.1556177e-001 Rv0020c Rv0494 -3.1271691e-001 Rv0020c Rv2175c 3.4868007e-001 Rv0020c Rv3414c 2.9117445e-001 Rv0020c Rv3676 3.1881544e-001 Rv0494 Rv2175c -3.4985986e-001 Rv0494 Rv3414c -2.6465222e-001 Rv0494 Rv3676 -2.2776261e-003 Rv2175c Rv3414c 4.4410537e-001 Rv2175c Rv3676 2.5027063e-001 Rv3414c Rv3676 4.4032942e-001 | = Rv0020 AND Rv0014 AND Rv3676 AND Rv2175 AND Rv3414OR NOT Rv0494 |
| Rv0179c | Rv0339c (0.512519) Rv2175c (0.540004) | Rv0339c Rv2175c 5.3693338e-001 | = Rv0339c AND Rv2175c |
| Rv0192 | Rv2175c (0.561563) | 0.56 | = Rv3414c |
| Rv0199 | Rv2175c (0.547938) | 0.55 | = Rv2175c |
| Rv0203 | Rv2175c (0.705253) | 0.71 | = Rv2175c |
| Rv0216 | Rv2175c (0.418089) | 0.42 | = Rv2175c |
| Rv0250c | Rv0020c (0.586706) Rv1221 (0.502351) Rv3223c (0.065707) | Rv0020c Rv1221 4.2770498e-001  Rv0020c Rv3223c 3.0347523e-001  Rv1221 Rv3223c 3.3184594e-001 | = Rv0020c AND Rv1221 AND Rv3223c |
| Rv0283 | Rv2069 (0.381658) Rv2175c (0.605676) Rv2359 (-0.140994) Rv2711 (-0.136149) Rv3260c (0.695643) Rv3414c (0.522951) | Rv2069 Rv2175c 3.1709964e-001 Rv2069 Rv2359 -1.2882920e-001 Rv2069 Rv2711 1.3891023e-001 Rv2069 Rv3260c 1.0498787e-001 Rv2069 Rv3414c -3.0127873e-003 Rv2175c Rv2359 1.6830468e-001 Rv2175c Rv2711 -1.3515796e-001 Rv2175c Rv3260c 6.7798935e-001 Rv2175c Rv3414c 4.4410537e-001 Rv2359 Rv2711 4.6115612e-001 Rv2359 Rv3260c -6.6590086e-002 Rv2359 Rv3414c -2.0271668e-001 Rv2711 Rv3260c -8.2505629e-002 Rv2711 Rv3414c -1.9623082e-001 Rv3260c Rv3414c 5.6861997e-001 | = Rv2069 OR (Rv3414 AND Rv3260 And Rv2175) OR (NOT Rv2711 AND NOT Rv2359) |
| Rv0287 | Rv2069 (0.352611) Rv2359 (-0.372696) Rv2711 (-0.136581) | Rv2069 Rv2359 -1.2882920e-001  Rv2069 Rv2711 1.3891023e-001  Rv2359 Rv2711 4.6115612e-001 | = Rv2069 OR (NOT Rv23359 AND Rv2711) |
| Rv0308 | Rv2027c (-0.046815) Rv3133c (0.050442) | Rv2027c Rv3133c 1.2017934e-001 | = Rv3133c OR NOT Rv2027c |
| Rv0339c | TF |  |  |
| Rv0350 | Rv0353 (0.438577) Rv3223c (0.079773) | Rv0353 Rv3223c 3.6715642e-002 | = Rv0353 OR Rv3223c |
| Rv0353 | Rv3223c (0.036716) | 0.04 | = Rv3223c |
| Rv0360c | Rv3260c (0.366246) Rv3286c (-0.117470) | Rv3260c Rv3286c -2.8796567e-002 | = Rv3260c OR NOT Rv3286c |
| Rv0429c | Rv0014c (0.378295) Rv3676 (0.170676) | Rv0014c Rv3676 4.1556177e-001 | = Rv0014c AND Rv3676 |
| Rv0430 | Rv2175c (0.660414) Rv3219 (0.645330) Rv3260c (0.721336) Rv3286c (-0.120379) | Rv2175c Rv3219 5.5073644e-001 Rv2175c Rv3260c 6.7798935e-001 Rv2175c Rv3286c 1.2661715e-001 Rv3219 Rv3260c 6.8467183e-001 Rv3219 Rv3286c 8.0116018e-002 Rv3260c Rv3286c -2.8796567e-002 | = Rv2175c AND Rv3219 AND Rv3260c OR NOT Rv3286 |
| Rv0431 | Rv0020c (0.579220) Rv3286c (-0.204143) | Rv0020c Rv3286c -1.0629458e-001 | = Rv0020c OR NOT Rv3286c |
| Rv0451c | Rv1343c (0.616831) Rv2711 (0.160042) Rv3676 (0.243590) | Rv1343c Rv2711 3.5346007e-002 Rv1343c Rv3676 1.5849879e-001 Rv2711 Rv3676 3.6994229e-002 | = Rv1343c OR Rv3676 OR NOT Rv2711 |
| Rv0479c | Rv2175c (0.630629) | 0.63 | = Rv2175c |
| Rv0482 | Rv2711 (-0.478178) | -0.48 | = NOT Rv2711 |
| Rv0483 | Rv3219 (0.611094) Rv3676 (0.346109) | Rv3219 Rv3676 4.4917928e-001 | = Rv3219 AND Rv3676 |
| Rv0485 | TF |  |  |
| Rv0491 | TF |  |  |
| Rv0494 | TF |  |  |
| Rv0502 | Rv0020c (0.621947) | 0.62 | = Rv0020c |
| Rv0511 | Rv0014c (0.638933) Rv0020c (0.565820) | Rv0014c Rv0020c 4.7334014e-001 | = Rv0014c AND Rv0020c |
| Rv0512 | Rv3246c (0.330764) | 0.33 | = Rv3246c |
| Rv0524 | Rv2711 (-0.355739) | -0.36 | = NOT Rv2711 |
| Rv0528 | Rv0014c (0.699851) Rv2711 (-0.358159) | Rv0014c Rv2711 -4.2333638e-002 | = Rv0014c AND NOT Rv2711 |
| Rv0543c | Rv0020c (0.630890) Rv2175c (0.544702) Rv3208 (0.721859) | Rv0020c Rv2175c 3.4868007e-001 Rv0020c Rv3208 5.9198510e-001 Rv2175c Rv3208 5.0414491e-001 | = Rv0020c AND Rv2175c AND Rv3208 |
| Rv0545c | Rv1626 (0.735399) | 0.74 | = Rv1626 |
| Rv0559c | Rv0020c (0.725380) | 0.73 | = Rv0020c |
| Rv0569 | Rv3132c (0.731045) | 0.73 | = Rv3132c |
| Rv0613c | Rv2175c (0.688978) | 0.69 | = Rv2175c |
| Rv0625c | Rv2175c (0.689260) | 0.69 | = Rv2175c |
| Rv0636 | Rv2175c (0.563394) Rv3219 (0.715617) | Rv2175c Rv3219 5.5073644e-001 | = Rv2175c AND Rv3219 |
| Rv0638 | Rv2175c (0.574839) Rv3219 (0.725547) Rv3260c (0.627158) | Rv2175c Rv3219 5.5073644e-001 Rv2175c Rv3260c 6.7798935e-001 Rv3219 Rv3260c 6.8467183e-001 | = Rv2175c AND Rv3219 AND Rv3260c |
| Rv0731c | Rv2175c (0.634140) | 0.63 | = Rv2175c |
| Rv0733 | Rv1626 (0.773407) | 0.77 | = Rv1626 |
| Rv0738 | Rv2175c (0.721748) Rv3414c (0.564701) | Rv2175c Rv3414c 4.4410537e-001 | = Rv2175c AND Rv3414c |
| Rv0756c | Rv0339c (0.656311) Rv2175c (0.635690) Rv3260c (0.696225) | Rv0339c Rv2175c 5.3693338e-001 Rv0339c Rv3260c 4.4169851e-001 Rv2175c Rv3260c 6.7798935e-001 | = Rv0339c AND Rv2175c AND Rv3260c |
| Rv0792c | TF |  |  |
| Rv0814c | Rv2175c (0.675148) Rv2621c (0.325230) Rv3219 (0.610575) Rv3260c (0.724604) | Rv2175c Rv2621c 2.6875631e-001 Rv2175c Rv3219 5.5073644e-001 Rv2175c Rv3260c 6.7798935e-001 Rv2621c Rv3219 2.5392193e-001 Rv2621c Rv3260c 4.1217065e-001 Rv3219 Rv3260c 6.8467183e-001 | = Rv2175c AND Rv3219 AND Rv3260c OR (Rv2621c AND Rv3260c) |
| Rv0815c | Rv2175c (0.698917) Rv3208 (0.578458) Rv3219 (0.748160) Rv3260c (0.716730) | Rv2175c Rv3208 5.0414491e-001 Rv2175c Rv3219 5.5073644e-001 Rv2175c Rv3260c 6.7798935e-001 Rv3208 Rv3219 3.8309911e-001 Rv3208 Rv3260c 5.2926209e-001 Rv3219 Rv3260c 6.8467183e-001 | = Rv3208 AND Rv2175c AND Rv3219 AND Rv3260c |
| Rv0844c | Rv0845c (0.415887) Rv2027c (0.31196179) Rv3132c (-0.073343) | Rv0845 Rv2027c 4.2203842e-001  Rv0845 Rv3132c 2.1857825e-001  Rv2027c Rv3132c 1.0967803e-001 | = Rv0845 AND Rv2027c OR NOT Rv3132c |
| Rv0845 | Rv0844c (0.415887) | 0.42 | = Rv0844c |
| Rv0854 | Rv3208 (0.489267) Rv3260c (0.465942) | Rv3208 Rv3260c 5.2926209e-001 | = Rv3208 AND Rv3260c |
| Rv0863 | Rv0020c (0.775471) Rv3219 (0.647814) | Rv0020c Rv3219 5.3258990e-001 | = Rv0020c AND Rv3219 |
| Rv0867c | Rv3676 (0.387899) | 0.39 | = Rv3676 |
| Rv0883c | Rv0020c (0.633828) Rv3208 (0.614619) Rv3260c (0.599499) Rv3295 (0.601787) | Rv0020c Rv3208 5.9198510e-001 Rv0020c Rv3260c 4.4154781e-001 Rv0020c Rv3295 5.2876279e-001 Rv3208 Rv3260c 5.2926209e-001 Rv3208 Rv3295 4.3041029e-001 Rv3260c Rv3295 5.4647348e-002 | = Rv0020c AND Rv3208 AND Rv 3295 AND Rv3260c |
| Rv0896 | Rv3676 (0.452525) | 0.45 | = Rv3676 |
| Rv0909 | Rv0020c (0.650827) | 0.65 | = Rv0020c |
| Rv0910 | Rv3208 (0.584411) | 0.58 | = Rv3208 |
| Rv0935 | Rv0491 (-0.449140) Rv1534 (0.741242) | Rv0491 Rv1534 -2.6359398e-001 | = Rv1534 OR NOT Rv0491 |
| Rv0954 | Rv0020c (0.557605) Rv2175c (0.706299) Rv3219 (0.672688) | Rv0020c Rv2175c 3.4868007e-001 Rv0020c Rv3219 5.3258990e-001 Rv2175c Rv3219 5.5073644e-001 | = Rv0020c And Rv2175c AND Rv3219 |
| Rv1002c | Rv3260c (0.458519) | 0.46 | = Rv3260c |
| Rv1018c | Rv0485 (0.240928) Rv3246c (0.608388) Rv3648c (0.594608) | Rv0485 Rv3246c 2.9916856e-001 Rv0485 Rv3648c 2.5313836e-001 Rv3246c Rv3648c 6.0129944e-001 | = Rv3246c AND Rv3648c OR Rv0485 |
| Rv1109c | Rv0020c (0.594483) Rv3295 (0.597165) | Rv0020c Rv3295 5.2876279e-001 | = Rv0020c AND Rv3295 |
| Rv1156 | Rv0020c (0.777647) Rv2175c (0.506093) | Rv0020c Rv2175c 3.4868007e-001 | = Rv0020c AND Rv2175c |
| Rv1158c | Rv3676 (0.334342) | 0.33 | = Rv3676 |
| Rv1166 | Rv0014c (0.744199) Rv3414c (0.558661) | Rv0014c Rv3414c 5.2640525e-001 | = Rv0014c AND Rv3414c |
| Rv1174c | Rv0020c (0.829841) | 0.83 | = Rv0020c |
| Rv1196 | Rv2359 (-0.335409) Rv3414c (0.560123) | Rv2359 Rv3414c -2.0271668e-001 | = Rv3414c OR NOT Rv2359 |
| Rv1211 | Rv3219 (0.534365) | 0.53 | = Rv3219 |
| Rv1221 | TF |  |  |
| Rv1280c | Rv0491 (-0.223031) Rv3291c (0.321199) | Rv0491 Rv3291c -4.0795646e-001 | = Rv3291c AND NOT Rv0491 |
| Rv1300 | Rv0014c (0.671035) Rv3295 (0.481311) | Rv0014c Rv3295 3.7640641e-001 | = Rv0014c AND Rv3295 |
| Rv1306 | Rv3219 (0.728535) Rv3648c (0.804444) | Rv3219 Rv3648c 6.2176231e-001 | = Rv3219 AND Rv3648c |
| Rv1307 | Rv1626 (0.819029) Rv3219 (0.659795) | Rv1626 Rv3219 6.4277476e-001 | = Rv1626 AND Rv3219 |
| Rv1308 | Rv3648c (0.813051) | 0.81 | = Rv3648c AND Rv3246c |
| Rv1309 | Rv3246c (0.475404) Rv3648c (0.803353) | Rv3246c Rv3648c 6.0129944e-001 | = Rv3246c AND Rv3648c |
| Rv1310 | Rv3246c (0.586315) Rv3648c (0.829276) | Rv3246c Rv3648c 6.0129944e-001 | = Rv3246c AND Rv3648c |
| Rv1311 | Rv0014c (0.760646) Rv1534 (0.754868) Rv3246c (0.554160) Rv3648c (0.767503) | Rv0014c Rv1534 6.7788369e-001 Rv0014c Rv3246c 4.0560816e-001 Rv0014c Rv3648c 5.9763877e-001 Rv1534 Rv3246c 4.2398120e-001 Rv1534 Rv3648c 5.2908124e-001 Rv3246c Rv3648c 6.0129944e-001 | = Rv0014c AND Rv1534 AND Rv3246c AND Rv3648c |
| Rv1312 | Rv0014c (0.805818) Rv3414c (0.570262) | Rv0014c Rv3414c 5.2640525e-001 | = Rv0014c AND Rv3414c |
| Rv1322 | Rv0020c (0.570517) Rv3219 (0.444275) | Rv0020c Rv3219 5.3258990e-001 | = Rv0020c AND Rv3219 |
| Rv1342c | Rv1343c (0.755709) Rv2711 (-0.086586) Rv3676 (-0.076554) | Rv1343c Rv2711 3.5346007e-002 Rv1343c Rv3676 1.5849879e-001 Rv2711 Rv3676 3.6994229e-002 | = Rv1343c OR NOT Rv2711 OR NOT Rv3676 |
| Rv1343c | Rv2711 (0.035346) Rv3676 (0.158499) | Rv2711 Rv3676 3.6994229e-002 | = Rv3676 OR Rv2711 |
| Rv1362c | Rv2175c (0.642127) | 0.64 | = Rv2175c |
| Rv1379 | Tf |  |  |
| Rv1387 | Rv3414c (0.369907) Rv3676 (0.358138) | Rv3414c Rv3676 4.4032942e-001 | = Rv3414c AND Rv3676 OR NOT Rv0494 |
| Rv1388 | Rv1626 (0.767899) Rv2175c (0.447217) | Rv1626 Rv2175c 6.3125387e-001 | = Rv1626 AND Rv2175c |
| Rv1395 | Tf |  |  |
| Rv1423 | Tf |  |  |
| Rv1436 | Rv1785c (-0.086303) Rv3676 (0.483045) | Rv1785c Rv3676 1.9037202e-002 | = Rv3676 OR NOT Rv1785c |
| Rv1437 | Rv1785c (0.185890) Rv3676 (0.284606) | Rv1785c Rv3676 1.9037202e-002 | = Rv1785c OR Rv3676 |
| Rv1438 | Rv1785c (0.060231) Rv3676 (0.331114) | Rv1785c Rv3676 1.9037202e-002 | = Rv1785c OR Rv3676 |
| Rv1462 | Rv1343c (0.731796) Rv2621c (0.715947) | Rv1343c Rv2621c 5.3811809e-001 | = Rv1343c AND Rv2621c |
| Rv1534 | TF |  |  |
| Rv1547 | Rv1423 (0.249616) Rv1626 (0.629287) | Rv1423 Rv1626 2.2305459e-001 | = Rv1626 OR Rv1423 |
| Rv1558 | Rv0019c (0.646298) Rv2175c (0.651284) Rv3291c (0.134621) | Rv0019c Rv2175c 5.0713406e-001 Rv0019c Rv3291c -1.0689887e-001 Rv2175c Rv3291c -3.7023671e-002 | = Rv0019c AND Rv2175c OR Rv3291c |
| Rv1565c | Rv0014c (0.757156) | 0.76 | = Rv0014c |
| Rv1590 | Rv2175c (0.518492) | 0.52 | = Rv2175c |
| Rv1592c | Rv1343c (0.468978) Rv3676 (0.062164) | Rv1343c Rv3676 1.5849879e-001 | = Rv1343c OR Rv3676 |
| Rv1613 | Rv0792c (-0.613622) Rv1534 (0.759890) Rv1626 (0.644244) | Rv0792c Rv1534 -3.5119997e-001 Rv0792c Rv1626 -4.8067323e-001 Rv1534 Rv1626 4.9253521e-001 | = Rv1534 AND Rv1626 AND (NOT Rv0792c) |
| Rv1614 | Rv0014c (0.764889) Rv1626 (0.630321) Rv1746 (0.634811) | Rv0014c Rv1626 5.5731056e-001 Rv0014c Rv1746 4.9565849e-001 Rv1626 Rv1746 2.3688669e-001 | = Rv0014c AND Rv1626 And Rv1746 |
| Rv1626 | Rv0015c (0.356596) | 0.36 | = Rv0015c |
| Rv1630 | Rv1423 (0.261209) Rv1626 (0.747040) | Rv1423 Rv1626 2.2305459e-001 | = Rv1630 OR Rv1423 |
| Rv1632c | Rv0014c (0.730986) | 0.73 | = Rv1423 |
| Rv1642 | Rv3246c (0.639443) | 0.64 | = Rv0014c |
| Rv1643 | Rv1626 (0.737026) Rv3246c (0.603677) | Rv1626 Rv3246c 6.7082620e-001 | = Rv1626 AND Rv3246c |
| Rv1697 | Rv0014c (0.715559) Rv2720 (0.302988) | Rv0014c Rv2720 1.2111201e-001 | = Rv0014c OR Rv2720 |
| Rv1698 | Rv0014c (0.696816) Rv2720 (0.151842) | Rv0014c Rv2720 1.2111201e-001 | = Rv0014c OR Rv2720 |
| Rv1700 | Rv0014c (0.513986) Rv3648c (0.580448) | Rv0014c Rv3648c 5.9763877e-001 | = Rv0014c AND Rv3648c |
| Rv1710 | Rv0014c (0.605927) Rv1534 (0.768547) | Rv0014c Rv1534 6.7788369e-001 | = Rv0014c AND Rv1534 |
| Rv1711 | Rv1746 (0.651817) | 0.65 | = Rv1746 |
| Rv1712 | Rv0844c (0.760001) Rv1534 (0.769062) | Rv0844c Rv1534 6.4360259e-001 | = Rv0844c AND Rv1534 |
| Rv1713 | Rv0353 (-0.290641) Rv0844c (0.640332) Rv1746 (0.735168) | Rv0353 Rv0844c -1.8157034e-001 Rv0353 Rv1746 -1.8683922e-001 Rv0844c Rv1746 4.7955561e-001 | = Rv1746 AND Rv0844c OR Rv0353 |
| Rv1736c | Rv3133c (0.755516) | 0.76 | = Rv3133c |
| Rv1737c | Rv0015c (-0.167281) Rv1626 (-0.004415) Rv3133c (0.741636) | Rv0015c Rv1626 3.5659598e-001 Rv0015c Rv3133c -1.7175603e-001 Rv1626 Rv3133c -1.6295340e-001 | = Rv3133c OR (NOT Rv0015c AND NOT Rv1626) |
| Rv1746 | Rv0014c (0.495658) | 0.5 | = Rv0014c |
| Rv1784 | Rv0014c (0.551570) Rv0020c (0.403115) | Rv0014c Rv0020c 4.7334014e-001 | = Rv0014c AND Rv0020c |
| Rv1785c | TF |  |  |
| Rv1789 | Rv0339c (0.448491) Rv2175c (0.660770) | Rv0339c Rv2175c 5.3693338e-001 | = Rv0339c AND Rv2175c |
| Rv1796 | Rv0020c (0.561440) Rv3295 (0.691362) | Rv0020c Rv3295 5.2876279e-001 | = Rv0020c AND Rv3295 |
| Rv1828 | Rv0020c (0.757895) | 0.76 | = Rv0020c |
| Rv1871c | Rv0020c (0.555656) Rv2359 (-0.302531) | Rv0020c Rv2359 6.2987745e-002 | = Rv0020c OR NOT Rv2359 |
| Rv1884c | Rv0020c (0.566398) | 0.57 | = Rv0020c |
| Rv1886c | Rv3219 (0.670085) | 0.67 | = Rv3219 |
| Rv1887 | Rv2175c (0.675780) | 0.68 | = Rv2175c |
| Rv1891 | Rv0020c (0.637300) Rv0339c (0.577570) Rv2175c (0.600147) | Rv0020c Rv0339c 3.8564153e-001 Rv0020c Rv2175c 3.4868007e-001 Rv0339c Rv2175c 5.3693338e-001 | = Rv0020c AND Rv0339c AND Rv2175 |
| Rv1980c | Rv0020c (0.584471) | 0.58 | = Rv0020c |
| Rv1984c | Rv0020c (0.365486) Rv0339c (0.344592) | Rv0020c Rv0339c 3.8564153e-001 | = Rv0020c AND Rv0339c |
| Rv1985c | TF |  |  |
| Rv1996 | Rv3133c (0.835896) | 0.84 | = Rv3133c |
| Rv1997 | Rv3133c (0.905632) | 0.91 | = Rv3133c |
| Rv2005c | Rv3132c (0.716617) Rv3133c (0.896391) | Rv3132c Rv3133c 8.6614612e-001 | = Rv3132c AND Rv3133c |
| Rv2006 | Rv3132c (0.787631) | 0.79 | = Rv3132c |
| Rv2007c | Rv2621c (0.282401) Rv3133c (0.817248) | Rv2621c Rv3133c 3.5432333e-001 | = Rv3133c AND Rv2621c |
| Rv2027c | Rv0844c (0.311962) | 0.31 | = Rv0844c |
| Rv2028c | Rv1343c (0.608200) Rv2027c (0.506251) Rv2069 (0.004885) Rv3132c (0.648575) | Rv1343c Rv2027c 3.1354733e-001 Rv1343c Rv2069 5.5772403e-002 Rv1343c Rv3132c 4.5276273e-001 Rv2027c Rv2069 -2.9982709e-001 Rv2027c Rv3132c 1.0967803e-001 Rv2069 Rv3132c 1.0934255e-002 | = Rv1343 AND Rv3132c AND Rv2027c OR Rv2069 |
| Rv2029c | Rv1379 (0.206805) Rv2069 (-0.028250) Rv3132c (0.671739) Rv3133c (0.807363) | Rv1379 Rv2069 2.9184784e-001 Rv1379 Rv3132c 3.5529649e-002 Rv1379 Rv3133c 2.2424428e-001 Rv2069 Rv3132c 1.0934255e-002 Rv2069 Rv3133c 1.8709063e-002 Rv3132c Rv3133c 8.6614612e-001 | = Rv3132c AND Rv3133c OR Rv1379 OR NOT RV2069 |
| Rv2030c | Rv2069 (-0.009448) Rv3132c (0.756509) Rv3133c (0.871921) | Rv2069 Rv3132c 1.0934255e-002  Rv2069 Rv3133c 1.8709063e-002  Rv3132c Rv3133c 8.6614612e-001 | = Rv3132c AND Rv3133c OR NOT RV2069 |
| Rv2031c | Rv2069 (0.024535) Rv3133c (0.871622) | Rv2069 Rv3133c 1.8709063e-002 | = Rv3133c OR NOT Rv2069 |
| Rv2032 | Rv3132c (0.659775) | 0.66 | = Rv3132c |
| Rv2036 | Rv2711 (0.379745) | 0.38 | = Rv2711 |
| Rv2049c | Rv2175c (0.606670) | 0.61 | = Rv2175c |
| Rv2061c | Rv2175c (0.643359) | 0.64 | = Rv2175c |
| Rv2069 | TF |  |  |
| Rv2074 | Rv1626 (0.750882) Rv3414c (0.648584) | Rv1626 Rv3414c 5.0951467e-001 | = Rv1626 AND Rv314c |
| Rv2091c | Rv0020c (0.724249) Rv2175c (0.499700) | Rv0020c Rv2175c 3.4868007e-001 | = Rv0020c AND Rv2175c |
| Rv2097c | Rv2175c (0.444122) Rv3219 (0.473123) | Rv2175c Rv3219 5.5073644e-001 | = Rv2175c AND Rv3219 |
| Rv2108 | Rv1343c (0.434059) | 0.43 | = Rv1343c |
| Rv2109c | Rv0014c (0.543368) Rv0019c (0.333301) Rv0020c (0.542453) Rv0339c (0.401397) | Rv0014c Rv0019c 5.6908563e-002 Rv0014c Rv0020c 4.7334014e-001 Rv0014c Rv0339c 2.2274579e-001 Rv0019c Rv0020c 4.2907013e-001 Rv0019c Rv0339c 4.7037777e-001 Rv0020c Rv0339c 3.8564153e-001 | = Rv0020c AND Rv0339 AND (Rv0014c OR Rv0019c) |
| Rv2112c | Rv2175c (0.525277) | 0.53 | = Rv2175c |
| Rv2123 | Rv1343c (0.562488) Rv2711 (0.082594) | Rv1343c Rv2711 3.5346007e-002 | = Rv1343 OR Rv2711 |
| Rv2145c | Rv0014c (0.591268) Rv0020c (0.630697) | Rv0014c Rv0020c 4.7334014e-001 | = Rv0014c AND Rv0020c |
| Rv2147c | Rv0020c (0.624949) Rv3246c (0.509127) | Rv0020c Rv3246c 6.2985804e-001 | = Rv0020c AND Rv3246c |
| Rv2149c | Rv3246c (0.448880) | 0.45 | = Rv3246c |
| Rv2163c | Rv0014c (0.514976) Rv0020c (0.577834) | Rv0014c Rv0020c 4.7334014e-001 | = Rv0014c AND Rv0020c |
| Rv2175c | Rv3260c (0.67798935) | 0.68 | = Rv2175c AND Rv3219 AND Rv3246c |
| Rv2184c | Rv2175c (0.564142) | 0.56 | = Rv2175c |
| Rv2194 | Rv0014c (0.614927) | 0.61 | = Rv0014c |
| Rv2198c | Rv1395 (-0.141900) Rv3414c (0.555662) | Rv1395 Rv3414c -1.3172892e-001 | = Rv3414c AND NOT Rv1395 |
| Rv2203 | Rv2175c (0.576408) | 0.58 | = Rv2175c |
| Rv2224c | Rv2175c (0.424617) | 0.42 | = Rv2175c |
| Rv2237 | Rv2175c (0.486117) | 0.49 | = Rv2175c |
| Rv2239c | Rv3676 (0.510836) | 0.51 | = Rv3676 |
| Rv2256c | Rv0019c (0.462380) Rv0020c (0.610389) Rv0339c (0.530557) | Rv0019c Rv0020c 4.2907013e-001 Rv0019c Rv0339c 4.7037777e-001 Rv0020c Rv0339c 3.8564153e-001 | = Rv0019c AND Rv0020c AND Rv0339c |
| Rv2286c | Rv0339c (0.575656) Rv2175c (0.739538) | Rv0339c Rv2175c 5.3693338e-001 | = Rv0339c AND Rv2175c |
| Rv2302 | Rv3219 (0.639451) | 0.64 | = Rv3219 |
| Rv2335 | Rv0844c (0.657905) Rv1534 (0.695669) | Rv0844c Rv1534 6.4360259e-001 | = Rv0844c AND Rv1534 |
| Rv2359 | TF |  |  |
| Rv2362c | Rv0014c (0.637485) | 0.64 | = Rv0014c |
| Rv2376c | Rv2175c (0.555041) | 0.56 | = Rv2175c |
| Rv2381c | Rv1343c (0.669785) Rv2711 (-0.258695) | Rv1343c Rv2711 3.5346007e-002 | = Rv1343c AND NOT Rv2711 |
| Rv2382c | Rv2711 (-0.399615) | -0.4 | = NOT Rv2711 |
| Rv2383c | Rv2711 (-0.335880) | -0.34 | = NOT Rv2711 |
| Rv2413c | Rv2175c (0.494794) | 0.49 | = Rv2175c |
| Rv2450c | Rv2175c (0.603027) Rv3260c (0.499325) | Rv2175c Rv3260c 6.7798935e-001 | = Rv2175c AND Rv3260c |
| Rv2457c | Rv1626 (0.793993) Rv3246c (0.614713) Rv3648c (0.814330) | Rv1626 Rv3246c 6.7082620e-001 Rv1626 Rv3648c 8.1381021e-001 Rv3246c Rv3648c 6.0129944e-001 | = Rv1626 AND Rv3246c AND Rv3648c |
| Rv2462c | Rv1626 (0.730676) Rv3246c (0.530612) | Rv1626 Rv3246c 6.7082620e-001 | = Rv1626 AND Rv3246c |
| Rv2468c | Rv2175c (0.582502) Rv3219 (0.709340) Rv3260c (0.646282) | Rv2175c Rv3219 5.5073644e-001 Rv2175c Rv3260c 6.7798935e-001 Rv3219 Rv3260c 6.8467183e-001 | = Rv2175c AND Rv3219 AND Rv3260c |
| Rv2507 | Rv0020c (0.653528) | 0.65 | = Rv0020c |
| Rv2619c | Rv1343c (0.731367) Rv2621c (0.702823) | Rv1343c Rv2621c 5.3811809e-001 | = Rv1343c AND Rv2621c |
| Rv2620c | Rv1343c (0.618724) Rv2621c (0.778991) | Rv1343c Rv2621c 5.3811809e-001 | = Rv1343c AND Rv2621c |
| Rv2621c | TF |  |  |
| Rv2626c | Rv0014c (-0.385147) Rv0015c (-0.054328) Rv0019c (-0.108831) | Rv0014c Rv0015c 4.9317619e-001 Rv0014c Rv0019c 5.6908563e-002 Rv0015c Rv0019c 3.9021494e-001 | = NOT Rv0014c AND NOT Rv0015c OR NOT Rv0019c |
| Rv2629 | Rv2621c (0.523143) Rv3132c (0.638436) | Rv2621c Rv3132c 1.8737765e-001 | = Rv2621c OR Rv3132c |
| Rv2672 | Rv2175c (0.526688) | 0.53 | = Rv2175c |
| Rv2696c | Rv2175c (0.435744) | 0.44 | = Rv2175c |
| Rv2697c | Rv3208 (0.336826) | 0.34 | = Rv3208 |
| Rv2708c | Rv2175c (0.572412) Rv3260c (0.601022) | Rv2175c Rv3260c 6.7798935e-001 | = Rv2175c AND Rv3260c |
| Rv2711 | Rv1343c (0.035346) | 0.04 | = 'Rv1343c |
| Rv2720 | TF |  |  |
| Rv2721c | Rv2175c (0.401262) Rv3260c (0.642643) | Rv2175c Rv3260c 6.7798935e-001 | = Rv2175c AND Rv3260c |
| Rv2783c | Rv1626 (0.695094) | 0.7 | = Rv1626 |
| Rv2840c | Rv0020c (0.779090) Rv2175c (0.422983) Rv3219 (0.729054) | Rv0020c Rv2175c 3.4868007e-001 Rv0020c Rv3219 5.3258990e-001 Rv2175c Rv3219 5.5073644e-001 | = Rv0020c And Rv2175c AND Rv3219 |
| Rv2860c | Rv1534 (0.797976) Rv3676 (0.064204) | Rv1534 Rv3676 1.5045557e-001 | = Rv1534 OR Rv3676 |
| Rv2864c | Rv2711 (-0.383069) Rv3133c (0.037793) | Rv2711 Rv3133c -1.2416755e-001 | = NOT Rv2711 OR Rv3133c |
| Rv2890c | Rv0014c (0.146560) Rv3246c (0.556160) | Rv0014c Rv3246c 4.0560816e-001 | = Rv0014c AND Rv3246c |
| Rv2902c | Rv0844c (0.159745) Rv0845 (0.045307) | Rv0844c Rv0845 4.1588735e-001 | = Rv0844c AND Rv0845 |
| Rv2909c | Rv1395 (-0.110303) Rv2069 (0.288731) Rv3219 (0.665994) | Rv1395 Rv2069 -1.8763477e-001 Rv1395 Rv3219 -1.3793316e-001 Rv2069 Rv3219 3.1596823e-001 | = Rv3219 AND Rv2069 OR NOT Rv1395 |
| Rv2948c | Rv0844c (0.526221) Rv1534 (0.744492) | Rv0844c Rv1534 6.4360259e-001 | = Rv0844c AND Rv1543 |
| Rv2966c | Rv1626 (-0.620048) | -0.62 | = NOT Rv1626 |
| Rv2985 | Rv0020c (0.631217) | 0.63 | = Rv0020c |
| Rv2986c | Rv0353 (0.144782) Rv3246c (0.702597) Rv3676 (0.229426) | Rv0353 Rv3246c 2.9407482e-001 Rv0353 Rv3676 1.6049609e-001 Rv3246c Rv3676 1.6829894e-001 | = Rv3246c OR Rv0353 OR Rv3676 |
| Rv3004 | Rv0019c (0.635795) Rv0339c (0.542194) Rv2175c (0.570028) | Rv0019c Rv0339c 4.7037777e-001 Rv0019c Rv2175c 5.0713406e-001 Rv0339c Rv2175c 5.3693338e-001 | = Rv0019c AND Rv0339c AND Rv2175c |
| Rv3018c | Rv1343c (0.484141) | 0.48 | = Rv1343c |
| Rv3019c | Rv1343c (0.482733) Rv2359 (-0.227135) | Rv1343c Rv2359 1.9569968e-001 | = Rv1343c OR NOT Rv2359 |
| Rv3024c | Rv1626 (0.690578) Rv3246c (0.607528) | Rv1626 Rv3246c 6.7082620e-001 | = Rv1626 AND Rv3246c |
| Rv3028c | Rv3676 (0.400914) | 0.4 | = Rv3676 |
| Rv3029c | Rv3676 (0.394146) | 0.39 | = Rv3677 |
| Rv3046c | Rv2175c (0.579563) | 0.58 | = Rv2175c |
| Rv3054c | Rv2027c (0.040569) Rv3133c (0.227558) Rv3223c (0.365161) | Rv2027c Rv3133c 1.2017934e-001 Rv2027c Rv3223c -2.1353879e-001 Rv3133c Rv3223c -3.6399703e-002 | = Rv2027c OR Rv3133c OR Rv3223c |
| Rv3118 | Rv2175c (0.631349) Rv3219 (0.578193) Rv3260c (0.681663) | Rv2175c Rv3219 5.5073644e-001 Rv2175c Rv3260c 6.7798935e-001 Rv3219 Rv3260c 6.8467183e-001 | = Rv2175c AND Rv3219 AND Rv3260c |
| Rv3125c | Rv1343c (0.445024) Rv2027c (0.599605) | Rv1343c Rv2027c 3.1354733e-001 | = Rv1343c AND Rv2027c |
| Rv3127 | Rv1343c (0.589312) Rv3132c (0.691425) Rv3133c (0.858861) | Rv1343c Rv3132c 4.5276273e-001 Rv1343c Rv3133c 5.5037408e-001 Rv3132c Rv3133c 8.6614612e-001 | = Rv3143c AND Rv3132c AND Rv3133c |
| Rv3129 | Rv3132c (0.684535) | 0.68 | = Rv3132c |
| Rv3130c | Rv3132c (0.606000) Rv3133c (0.807565) | Rv3132c Rv3133c 8.6614612e-001 | = Rv3132c AND Rv3133c |
| Rv3131 | Rv1343c (0.581975) Rv3132c (0.677269) Rv3133c (0.815389) | Rv1343c Rv3132c 4.5276273e-001  Rv1343c Rv3133c 5.5037408e-001  Rv3132c Rv3133c 8.6614612e-001 | = Rv3132c AND Rv3133c AND Rv1343c_0 |
| Rv3132c | Rv0081 (0.711794) Rv0844c (-0.073343) Rv3133c (0.866146) | Rv0081 Rv0844c -7.1715707e-003  Rv0081 Rv3133c 7.4928957e-001  Rv0844c Rv3133c -2.5455257e-001 | = Rv0081 AND Rv3133c OR NOT Rv0844c |
| Rv3133c | Rv0845 (0.195179) Rv2027c (0.120179) | Rv0845 Rv2027c 4.2203842e-001 | = Rv0845 AND rv2027c |
| Rv3134c | Rv3132c (0.637223) Rv3133c (0.836030) | Rv3132c Rv3133c 8.6614612e-001 | = Rv3133c |
| Rv3208 | Rv3246c (0.448880) | 0.45 | ='Rv3246c' |
| Rv3219 | Rv3676 (0.449179) | 0.68 | = Rv3676c |
| Rv3223c | TF |  |  |
| Rv3246c | Rv1626 (0.670826) Rv3208 (0.715669) | Rv1626 Rv3208 5.8051376e-001 | = Rv1626 AND Rv3208 |
| Rv3248c | Rv3246c (0.668497) | 0.67 | = Rv3246c |
| Rv3257c | Rv3208 (0.677925) | 0.68 | = Rv3208 |
| Rv3258c | Rv1626 (0.717840) Rv2175c (0.627510) Rv3208 (0.567094) Rv3219 (0.545557) Rv3246c (0.815994) Rv3260c (0.812180) Rv3414c (0.693532) | Rv1626 Rv2175c 6.3125387e-001 Rv1626 Rv3208 5.8051376e-001 Rv1626 Rv3219 6.4277476e-001 Rv1626 Rv3246c 6.7082620e-001 Rv1626 Rv3260c 6.3925748e-001 Rv1626 Rv3414c 5.0951467e-001 Rv2175c Rv3208 5.0414491e-001 Rv2175c Rv3219 5.5073644e-001 Rv2175c Rv3246c 5.5545375e-001 Rv2175c Rv3260c 6.7798935e-001 Rv2175c Rv3414c 4.4410537e-001 Rv3208 Rv3219 3.8309911e-001 Rv3208 Rv3246c 7.1566901e-001 Rv3208 Rv3260c 5.2926209e-001 Rv3208 Rv3414c 3.8224195e-001 Rv3219 Rv3246c 4.6971421e-001 Rv3219 Rv3260c 6.8467183e-001 Rv3219 Rv3414c 4.0361233e-001 Rv3246c Rv3260c 7.4875790e-001 Rv3246c Rv3414c 5.4195881e-001 Rv3260c Rv3414c 5.6861997e-001 | = Rv1626 AND Rv2175c AND Rv3208 AND Rv3219 AND Rv3246c AND Rv3260c AND Rv3414c |
| Rv3259 | Rv3260c (0.445109) | 0.45 | = Rv3260c |
| Rv3260c | Rv2175c (0.677989) Rv3219 (0.684672) Rv3246c (0.748758) | Rv2175c Rv3219 5.5073644e-001  Rv2175c Rv3246c 5.5545375e-001  Rv3219 Rv3246c 4.6971421e-001 | = Rv2175c AND Rv3219 AND Rv3246c |
| Rv3267 | Rv0014c (0.484207) Rv2069 (-0.025368) | Rv0014c Rv2069 2.2259273e-001 | = Rv0014c OR NOT Rv2069 |
| Rv3277 | Rv3208 (0.546317) | 0.55 | = Rv3208 |
| Rv3278c | Rv3208 (0.575970) | 0.58 | = Rv3208 |
| Rv3286c | TF |  |  |
| Rv3291c | TF |  |  |
| Rv3295 | TF |  |  |
| Rv3317 | Rv3260c (0.541482) | 0.54 | = Rv3260c |
| Rv3332 | Rv0485 (-0.174027) Rv0792c (0.093509) Rv3676 (-0.064313) | Rv0485 Rv0792c 1.8364711e-001  Rv0485 Rv3676 4.3168673e-002  Rv0792c Rv3676 -1.6597244e-001 | = Rv0792c OR NOT Rv0485 OR NOT Rv3676 |
| Rv3346c | Rv0339c (0.653175) Rv2175c (0.719515) | Rv0339c Rv2175c 5.3693338e-001 | = Rv0339c AND Rv2175c |
| Rv3409c | Rv3219 (0.599658) Rv3414c (0.669536) | Rv3219 Rv3414c 4.0361233e-001 | = Rv3219 AND Rv3414c |
| Rv3414c | TF |  |  |
| Rv3489 | Rv0020c (0.736811) Rv2711 (0.177686) Rv3208 (0.710047) | Rv0020c Rv2711 4.4366459e-001  Rv0020c Rv3208 5.9198510e-001  Rv2711 Rv3208 1.7399599e-001 | = Rv0020c AND Rv3208 OR Rv2711 |
| Rv3493c | Rv3414c (0.557152) Rv3574 (-0.508309) | Rv3414c Rv3574 -3.5948130e-001 | = Rv3414c AND NOT Rv3574 |
| Rv3496c | Rv3414c (0.588241) Rv3574 (-0.420386) | Rv3414c Rv3574 -3.5948130e-001 | = Rv3414c AND NOT Rv3574 |
| Rv3574 | TF |  |  |
| Rv3587c | Rv0020c (0.568512) Rv0339c (0.425480) Rv3260c (0.712202) Rv3414c (0.627543) | Rv0020c Rv0339c 3.8564153e-001 Rv0020c Rv3260c 4.4154781e-001 Rv0020c Rv3414c 2.9117445e-001 Rv0339c Rv3260c 4.4169851e-001 Rv0339c Rv3414c 3.1185961e-001 Rv3260c Rv3414c 5.6861997e-001 | = Rv0020c AND Rv0339c AND Rv3260c AND Rv3414c |
| Rv3592 | Rv0020c (0.721988) Rv3208 (0.744912) | Rv0020c Rv3208 5.9198510e-001 | = Rv0020c AND Rv3208 |
| Rv3597c | Rv0020c (0.560193) Rv3219 (0.667051) Rv3260c (0.633346) | Rv0020c Rv3219 5.3258990e-001  Rv0020c Rv3260c 4.4154781e-001  Rv3219 Rv3260c 6.8467183e-001 | = Rv0020c AND Rv3219 AND Rv3260c |
| Rv3646c | Rv0014c (0.388759) | 0.39 | = Rv0014c |
| Rv3648c | TF |  |  |
| Rv3651 | Rv2175c (0.457311) | 0.46 | = Rv2175c |
| Rv3676 | Rv1343c (0.158499), Rv3219 (0.449179) | Rv1343c Rv3219 3.7193967e-001 | = Rv1343c AND Rv3219 |
| Rv3682 | Rv0014c (0.700491) Rv3414c (0.573090) | Rv0014c Rv3414c 5.2640525e-001 | = Rv0014c AND Rv3414c |
| Rv3714c | Rv2175c (0.473885) Rv3260c (0.623463) | Rv2175c Rv3260c 6.7798935e-001 | = Rv2175c AND Rv3260c |
| Rv3717 | Rv0014c (0.726267) | 0.73 | = Rv0014c |
| Rv3782 | Rv0020c (0.550226) Rv3208 (0.690696) Rv3260c (0.572256) | Rv0020c Rv3208 5.9198510e-001  Rv0020c Rv3260c 4.4154781e-001  Rv3208 Rv3260c 5.2926209e-00 | = Rv0020c AND Rv3260c AND Rv3208 |
| Rv3811 | Rv0020c (0.641774) | 0.64 | = Rv0020c |
| Rv3847 | Rv2175c (0.514633) | 0.51 | = Rv2175c |
| Rv3849 | Rv0020c (0.525015) Rv2175c (0.649715) Rv3260c (0.727746) | Rv0020c Rv2175c 3.4868007e-001 Rv0020c Rv3260c 4.4154781e-001 Rv2175c Rv3260c 6.7798935e-001 | = Rv0020c AND Rv2175c AND Rv3260c |
| Rv3869 | Rv0014c (0.754392) Rv0020c (0.601383) | Rv0014c Rv0020c 4.7334014e-001 | = Rv0014c AND Rv0020c |
| Rv3870 | Rv0014c (0.745990) | 0.75 | = Rv0014c |
| Rv3871 | Rv0014c (0.637997) | 0.64 | = Rv0014c |
| Rv3875 | Rv0020c (0.683332) Rv3219 (0.746778) | Rv0020c Rv3219 5.3258990e-001 | = Rv0020c AND Rv3219 |
| Rv3876 | Rv0020c (0.527705) Rv3208 (0.621011) Rv3414c (0.598408) | Rv0020c Rv3208 5.9198510e-001 Rv0020c Rv3414c 2.9117445e-001 Rv3208 Rv3414c 3.8224195e-001 | = Rv0020c AND Rv3208 AND Rv3414c |
| Rv3910 | Rv0014c (0.731803) Rv3414c (0.511734) | Rv0014c Rv3414c 5.2640525e-001 | = Rv0014c And Rv3414c |
| Rv3913 | Rv0117 (0.021331) Rv3223c (0.204868) | Rv0117 Rv3223c -2.1214514e-002 | = Rv3223c OR NOT Rv0117 |
| Rv3914 | Rv0117 (-0.230363) Rv3223c (0.079725) | Rv0117 Rv3223c -2.1214514e-002 | = Rv3223c OR NOT Rv0117 |
| Rv3920c | Rv0014c (0.563272) | 0.56 | = Rv0014c |
| Rv3921c | Rv1626 (0.656195) Rv3246c (0.498023) | Rv1626 Rv3246c 6.7082620e-001 | = Rv1626 AND Rv3246c |
| Rv3923c | Rv0019c (0.567438) Rv0020c (0.523543) Rv2175c (0.622723) | Rv0019c Rv0020c 4.2907013e-001 Rv0019c Rv2175c 5.0713406e-001 Rv0020c Rv2175c 3.4868007e-001 | = Rv0019c AND Rv0020c AND Rv2175c |
